# Artificial Intelligence Methods for Diagnostic and Decision-Making Assistance in Chronic Wounds: A Systematic Review

**DOI:** 10.1007/s10916-025-02153-8

**Published:** 2025-02-19

**Authors:** David Reifs Jiménez, Lorena Casanova-Lozano, Sergi Grau-Carrión, Ramon Reig-Bolaño

**Affiliations:** Digital Care Research Group, University of Vic, C/ Sagrada Familia, 7, 08500 Vic, Barcelona Spain

**Keywords:** Wounds and injuries, Algorithms, data mining, Deep learning, Computer assisted, Decision support systems

## Abstract

Chronic wounds, which take over four weeks to heal, are a major global health issue linked to conditions such as diabetes, venous insufficiency, arterial diseases, and pressure ulcers. These wounds cause pain, reduce quality of life, and impose significant economic burdens. This systematic review explores the impact of technological advancements on the diagnosis of chronic wounds, focusing on how computational methods in wound image and data analysis improve diagnostic precision and patient outcomes. A literature search was conducted in databases including ACM, IEEE, PubMed, Scopus, and Web of Science, covering studies from 2013 to 2023. The focus was on articles applying complex computational techniques to analyze chronic wound images and clinical data. Exclusion criteria were non-image samples, review articles, and non-English or non-Spanish texts. From 2,791 articles identified, 93 full-text studies were selected for final analysis. The review identified significant advancements in tissue classification, wound measurement, segmentation, prediction of wound aetiology, risk indicators, and healing potential. The use of image-based and data-driven methods has proven to enhance diagnostic accuracy and treatment efficiency in chronic wound care. The integration of technology into chronic wound diagnosis has shown a transformative effect, improving diagnostic capabilities, patient care, and reducing healthcare costs. Continued research and innovation in computational techniques are essential to unlock their full potential in managing chronic wounds effectively.

**Supplementary Information:**

The online version contains supplementary material available at 10.1007/s10916-025-02153-8.

## Introduction

A chronic wound is a wound that fails to heal within the expected timeframe, typically beyond 4-6 weeks, and does not progress through the normal stages of healing (inflammatory, proliferative, and remodeling) [1, 2]. Chronic wounds remain in a stalled phase due to factors such as underlying medical conditions, poor blood supply, or inadequate treatment [3]. In contrast, a hard-to-heal wound refers to a wound that initially begins healing but fails to show significant reduction in size (20%-40%) within the first 2-4 weeks of optimal treatment [4, 5]. While hard-to-heal wounds may eventually become chronic if healing is not achieved over time, not all chronic wounds are initially hard-to-heal [6, 7]. The key difference lies in the time frame and stage of healing: chronic wounds are defined by prolonged healing beyond 6 weeks, while hard-to-heal wounds show resistance to healing early in the process. In either case, these wounds can result from various underlying factors, such as the age of the patient and the presence of underlying chronic comorbidities [8]. The prevalence of chronic wounds is more significant than most people realize, making it a silent epidemic [9]. According to the World Health Organization (WHO), an estimated 4.5 million people worldwide suffer from chronic wounds at any given time. This number is expected to rise due to the aging population, the increasing prevalence of conditions like diabetes and obesity, and a growing number of people with reduced mobility [10]. Diabetes, in particular, is a leading cause of chronic wounds. Diabetic foot ulcers (DFU) alone affect approximately 15% of individuals with diabetes during their lifetime, and it’s estimated that 1 in 4 diabetics will experience a foot ulcer at some point [11]. The impact of chronic wounds on patients’ lives cannot be underrated. Besides the physical pain and discomfort, chronic wounds often lead to emotional distress and decreased mental well-being. Patients may experience anxiety, depression, social isolation, and a loss of independence due to restricted mobility [1, 12]. The risk of infection is another significant concern with chronic wounds, as they create an entry point for bacteria and can lead to amputations, further exacerbating the physical and psychological burden on affected individuals [13].

Chronic wounds place a substantial financial burden on healthcare systems and society. Treatment costs for managing chronic wounds are high, often requiring specialized wound care products, prolonged hospital stays, and frequent follow-up visits [14]. It is often believed that the use of wound dressings per se is the major cost driver in wound management, whereas, in fact, nursing time and hospital costs are together responsible for around 80-85% of the total cost [8]. For a diabetic foot wound alone, the estimated cost of treatment is approximately 10,000 euros [15]. Additionally, chronic wounds can lead to decreased productivity and lost workdays for patients and caregivers. Wound management is estimated to account for over 50% of community nurse time in European studies, with patients often having three or more home health visits per week [1]. The cost of reduced quality of life, combined with the economic impact, underscores the urgency of finding effective prevention and treatment strategies.

Early detection and proper management are crucial in addressing chronic wounds effectively. A multidisciplinary approach that involves wound care specialists, physicians, nurses, dietitians, and other healthcare professionals is often necessary. Preventive measures are equally important, especially for high-risk individuals. For diabetics, strict glycemic control, foot care, and regular medical check-ups can significantly reduce the risk of developing chronic wounds. Proper nutrition, maintaining a healthy weight, and regular exercise can help prevent wounds caused by arterial and venous insufficiency [11].

The fight against chronic wounds is ongoing, and innovative approaches are emerging to improve patient outcomes. Researchers are exploring advanced technologies to transform various aspects of healthcare, including chronic wound diagnosis and management, and provide clinicians with user-friendly tools to capture wound images, measure dimensions, and track healing progress over time [16]. This data helps in tailoring personalized treatment plans and identifying potential complications early on. With Artificial Intelligence (AI) and/or Machine Learning (ML) algorithms, it is possible to analyze vast amounts of wound-related data, identify patterns, and assist clinicians in making informed decisions, including the early detection of infection, reducing the risk of complications [17]. Telemedicine and digital health solutions are also being utilized to monitor and manage chronic wounds remotely, making healthcare more accessible to patients, especially those in rural or underserved areas [18].

The aim of this systematic review was to compile and analyze all relevant studies and reports published between 2013 and 2023, specifically focusing on the application of AI methods in the diagnosis of chronic wounds. The review provides a comprehensive overview of the various technological approaches employed in this period, classifying them based on their functionalities, such as tissue classification, wound area measurement, image segmentation, wound classification, and healing prediction. This classification highlights the advancements in AI-driven techniques and evaluates the outcomes achieved through these methods in supporting chronic wound diagnosis and management.

## Methods

The review adhered to the PRISMA (Preferred Reporting Items for Systematic Reviews and Meta-Analyses) framework [19, 20], ensuring a structured and transparent approach to data collection and analysis. The search spanned five major databases: ACM, IEEE, PubMed, Scopus, and Web of Science. Only studies published between 2013 and 2023 focusing on wound diagnosis through AI or ML-based methods were included. Exclusion criteria included non-English and non-Spanish articles, reviews, and those not focusing on image- or clinical data-based wound analysis.

### Search Strategy

Advanced search queries included terms such as ‘wound’, ‘ulcer’, ‘skin lesion’ and ‘diagnosis’, combined with ‘artificial intelligence’, ‘machine learning’ and other related computational methods. Broader search terms were initially used, then the first 50 results were analysed and more specific terms were incorporated into advanced database searches, using Boolean operators (OR, AND) to refine and reduce the number of results. Articles were selected based on their relevance to chronic wound diagnosis, with a specific focus on studies using RGB images or clinical patient data and computational techniques such as deep learning and supervised learning.

### Selection Criteria

The inclusion criteria for this systematic review encompassed articles published between January 1, 2013, and May 1, 2023, that included in the title or abstract the following terms: (diagnosis OR ”follow up” OR monitoring OR healing OR assessment, treatment OR identification OR management OR evaluation OR recognition OR classification OR segmentation) AND (wounds OR ulcers OR ”skin lesions” OR ”skin damage”) AND (”artificial intelligence” OR ”machine learning” OR ”deep learning” OR ”convolutional neural network” OR ”deep neural network” OR ”supervised learning” OR ”transfer learning” OR ’delineation OR ”support vector machine” OR clustering OR ”automatic detection” OR ”computer assisted”).

Exclusion criteria were other types of non-superficial wounds and non RGB images such as thermography, X-ray, ultrasound, computed tomography, and magnetic resonance imaging. Skin lesions such as melanomas or skin cancer and burns with interesting methods have been screened to leave as "searchable" but were not included for analysis in this systematic search. However, articles that included this type of wounds along with chronic wounds, and whose objective fell within the scope of this systematic review, were also included for evaluation. Review articles, letters to editors or editorials, non-English and non-Spanish articles or articles without full texts are excluded.

### Data Extraction and Synthesis

Key data points were systematically extracted, including the title, authors, abstract, year of publication and article type. The synthesis focused on the clinical utility of AI in wound care, detailing the ML models employed and identifying challenges related to decision support in chronic wound diagnosis.

To minimize the risk of bias, three independent reviewers assessed the included studies. One reviewer analyzed all studies, while the other two split the remaining studies equally. Each study was evaluated for inclusion, with a clear justification provided for exclusions. In cases of disagreement, the reviewers engaged in discussion until a consensus was reached, ensuring the integrity and consistency of the review process.

The final stage of the analysis involved a thorough examination of the studies and reports selected based on the established inclusion and exclusion criteria. For each study, key information was synthesized, including the type of technology employed, validation methods used, the type of chronic wound addressed, the number of samples, and the outcomes reported. During this phase, some additional articles that were not excluded in the initial screening based on title and abstract were discarded, following a more detailed review of their content.

Additionally, three articles identified from local literature, which were deemed relevant to the objectives of the systematic review, were included in the final analysis. These articles provided additional insights and were integrated into the review alongside the studies selected through the formal database search.

## Results

The systematic search identified a total of 2,791 articles, distributed across five major databases: 33 articles from ACM, 94 from IEEE, 529 from PubMed, 1,258 from Scopus, and 877 from Web of Science. After removing 1,298 duplicate articles, 1,493 unique articles were selected for further analysis. The initial screening, based on title and abstract, led to the exclusion of 1,350 articles due to differing objectives or failure to meet the inclusion and exclusion criteria. This resulted in 143 reports for in-depth reading and evaluation.

**Fig. 1:**
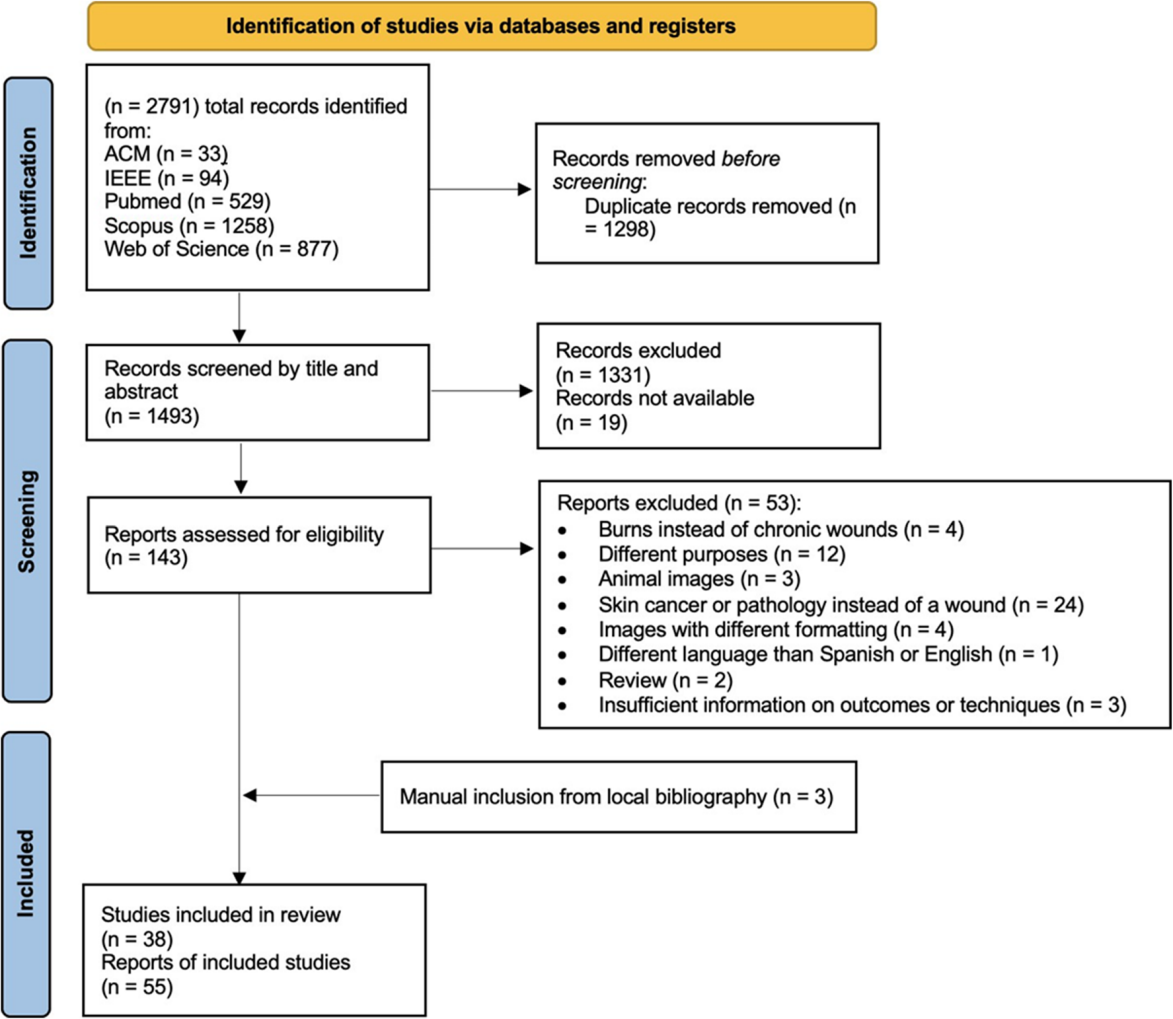
PRISMA Flow Diagram summarizing the selection process [19]

Of the 143 reports, 53 were excluded for various reasons: 4 focused on burns rather than chronic wounds, 12 had unrelated objectives, 3 utilized animal images, 24 were not related to chronic wounds (most were dermatoscopic images of skin cancer), 4 used advanced imaging techniques like computed tomography or X-ray instead of RGB images, 1 was not available in English or Spanish, 2 were review articles, and 3 did not give enough information on outcomes or techniques. After excluding these, a total of 90 articles remained. Additionally, three articles were manually added to these 90, bringing the final total to 93 articles included in this systematic review. These 93 articles comprise 38 studies and 55 reports focused on the development of specific technologies. The flow chart with the systematic research selection process is shown in Fig. 1.

### Characteristics of Included Articles

Of the 93 studies and final reports analyzed, 38 were based on clinical trials with real patients, and 55 were reports describing the methodology of technologies developed to support the diagnosis of various chronic wound types using images from databases. The most commonly addressed wound types include pressure ulcers (PU), diabetic foot ulcers (DFU), venous ulcers (VU), surgical wounds (SW), arterial ulcers (AU), and chronic wounds in general (CW).

Two types of samples are typically distinguished: in most cases, wound images are used, while in others, data from the patient’s medical history, test results, and analyses are utilized. In a few cases, both types of samples are combined. Articles using wound images often apply algorithms based on Deep Learning (DL) techniques, sometimes incorporating Computer-Vision (CV) techniques for image processing. In contrast, studies using patient data tend to apply ML algorithms.

**Fig. 2:**
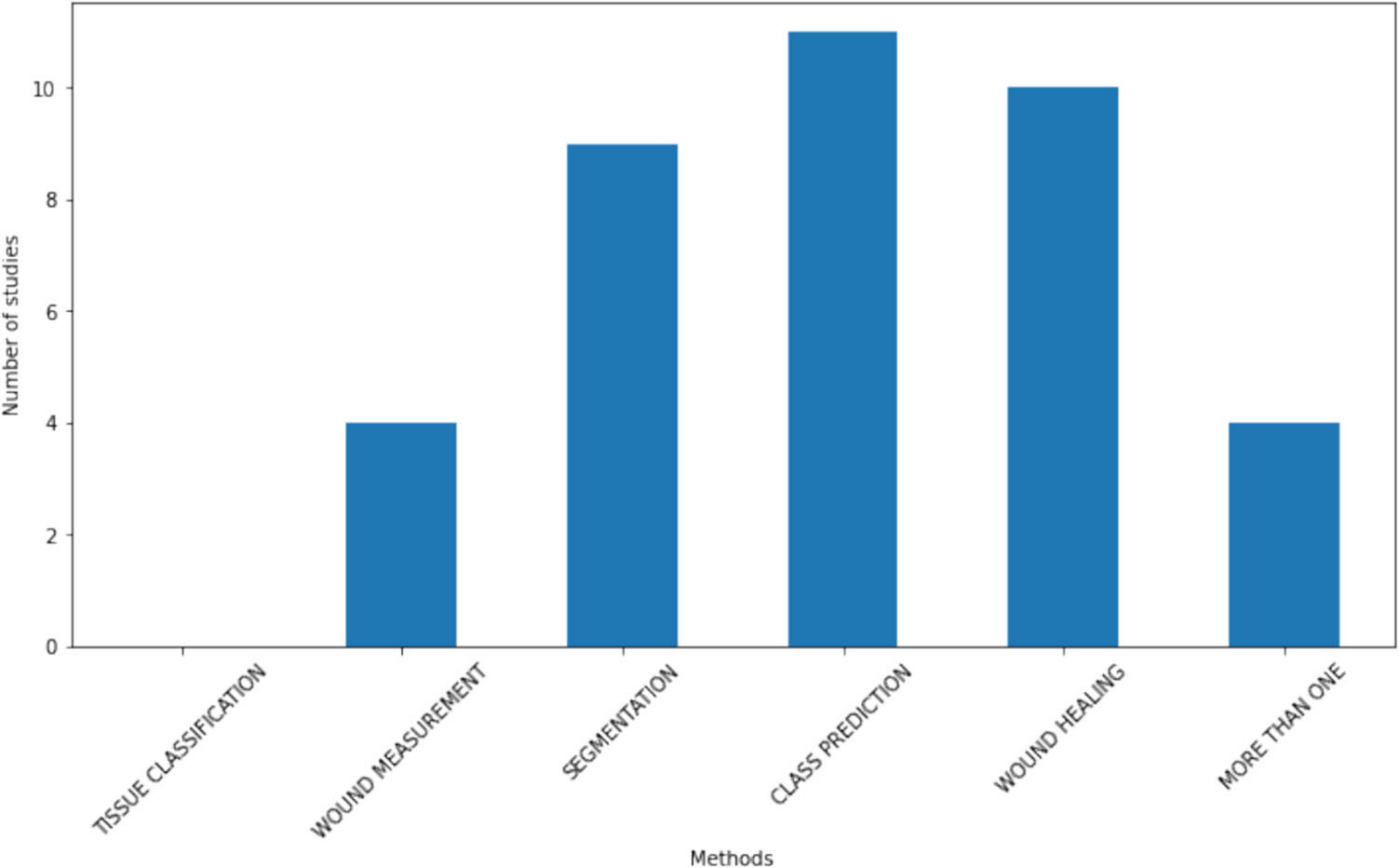
Task distribution between selected studies

**Fig. 3:**
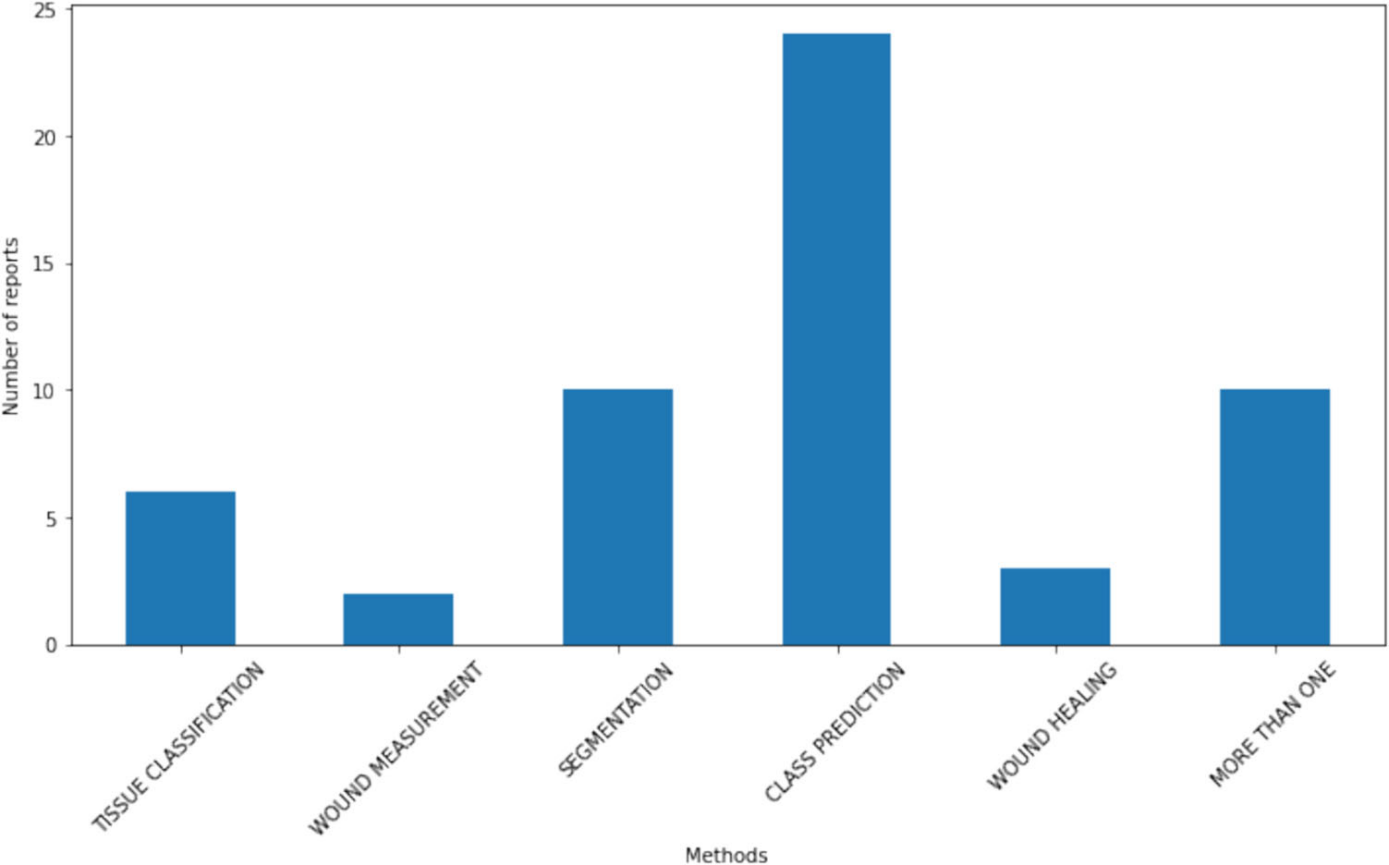
Task distribution between selected reports

One of the primary objectives of this systematic review is to identify and categorize the key methods used to support chronic wound diagnosis through AI or ML. The identified methods can be classified into several categories: Wound tissue classification techniques Section “Wound Tissue Classification”, wound measurement Section “Wound Measurement”, wound segmentation Section “Wound Segmentation”, class prediction (often focused on different etiologies) Section “Wound Classification”, and wound healing prediction or risk indicators Section “Wound Healing”. Notably, some articles were found to employ more than one of these tasks, demonstrating the versatility of AI and/or ML in addressing multiple aspects of wound diagnosis and management. Figures 2 and 3 show the distribution of tasks among the selected studies and reports, respectively. In total, 6 articles (0 studies, 6 reports) address tissue classification, 6 articles (4 studies, 2 reports) focus on wound measurement, 19 articles (9 studies, 10 reports) examine wound segmentation, 35 articles (11 studies, 24 reports) classify wounds mostly by etiology, and 13 articles (10 studies, 3 reports) predict wound healing. Additionally, 14 articles (4 studies, 10 reports) employ multiple previous tasks in their analyses Section “Multiple Methods”.

### Quality Assessment

A systematic evaluation was conducted to assess the methodological quality of 93 studies using the QUADAS-2 framework [21]. Although originally designed for clinical trials, QUADAS-2 evaluates the quality of methods and data related to diagnostic tools, making it applicable to studies that develop or validate diagnostic models, such as those employing machine learning or imaging technologies [22]. Many of the reviewed articles involved diagnostic processes where models were tested for accuracy, functioning similarly to diagnostic tools in clinical trials.

**Fig. 4:**
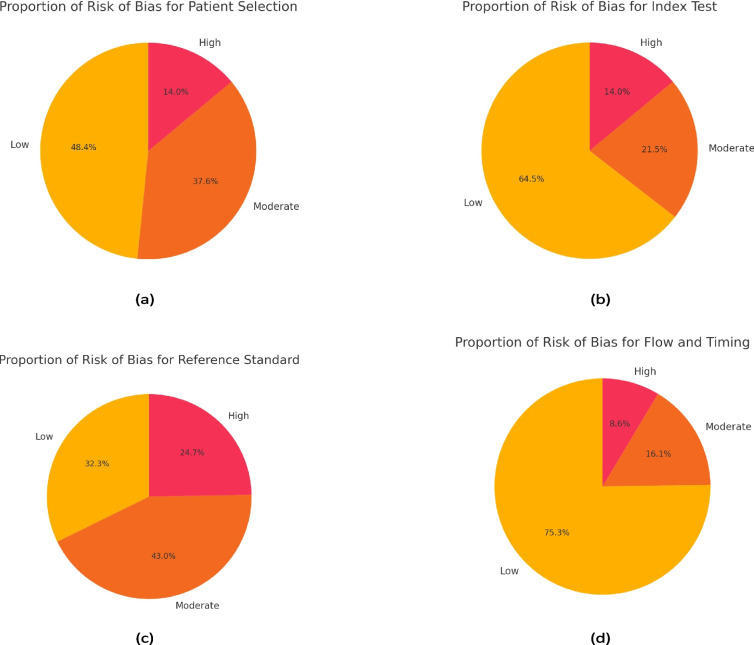
Proportion of articles with low, moderate and high risk of bias by a) Patient selection, b) Index Test, c) Reference Standard and d) Flow and Timing

The assessment included adaptations to align with the nature of non-clinical diagnostic studies. The Patient Selection domain was adjusted to evaluate the representativeness of datasets and the inclusion or exclusion criteria for data points, rather than focusing solely on patient-level factors. In the Index Test domain, the methodological rigor of the diagnostic models, including algorithms and neural networks, was examined, particularly their training and validation protocols. The Reference Standard domain was evaluated based on the quality and reliability of annotations or ground truth data, even when not derived from clinical settings. Flow and Timing was interpreted to assess the overall data handling, including exclusions and the completeness of the analysis process (Fig. 4).

In the domain of Patient Selection, most studies demonstrated a low risk of bias due to the use of clear inclusion criteria and appropriate sampling. However, around 25% showed moderate or high risk, often because of limited diversity in datasets or unrepresentative populations. Studies relying on datasets from single institutions or specific regions lacked broader demographic and clinical applicability. For the Index Test, the majority of studies exhibited low risk of bias, with clear documentation of machine learning methodologies and robust validation. Nevertheless, a minority had moderate risk due to inadequate blinding or reliance on pre-trained models without testing on independent datasets.

The Reference Standard domain had the highest proportion of moderate-to-high risk of bias. Many studies failed to adequately describe how ground truth labels were generated, with few employing blinded or independent expert validation. The absence of inter-rater reliability metrics or external validation raised concerns about the consistency and robustness of the reference standards. Flow and Timing presented the lowest risk of bias, with most studies showing well-documented workflows and minimal exclusions. However, some studies lacked clarity on the timing between data acquisition and reference standard application, potentially introducing bias.

Overall, studies utilizing large, diverse datasets with rigorous validation processes demonstrated consistently lower risk of bias. Conversely, innovative studies, such as those integrating 3D modeling or portable devices, often lacked robust external validation, contributing to moderate concerns, particularly in the Reference Standard domain. Across all domains, common limitations included insufficient diversity in patient demographics and a reliance on single-center datasets, which hindered generalizability.

**Table 1:** Key evaluation metrics used in chronic wound diagnosis with AI models

Metric	Formula	Definition
Accuracy	$Acc = \frac{TP + TN}{TP + TN + FP + FN}$	The proportion of correct predictions made by the model compared to the total number of instances
Recall, Sensitivity or True Positive Rate (TPR)	$SE = \frac{TP}{TP + FN}$	The proportion of true positives correctly identified by the model, crucial for ensuring that no important chronic wound cases are missed
Specificity	$SP = \frac{TN}{TN + FP}$	The proportion of actual negatives correctly identified, helping to measure how well the model avoids false positives
Precision or Positive Predictive Value (PPV)	$P = \frac{TP}{TP + FP}$	The proportion of true positive results in all predicted positives. Important when the cost of false positives is high
F1-Score	$P = 2*\frac{1}{\frac{1}{precision} + \frac{1}{recall}}$	The harmonic mean of precision and recall, providing a balance between the two. Useful in imbalanced datasets to ensure both false positives and false negatives are minimized
False Positive Rate (FPR)	$P = \frac{FP}{FP + TN}$	The rate of false positives in relation to actual negative cases, important for ensuring that the AI model does not incorrectly classify healthy tissue as problematic
Matthews Correlation Coefficient (MCC)	MCC = (TP * TN - FP * FN) / $\surd$((TP + FP) * (TP + FN) * (TN + FP) * (TN + FN))	A balanced measure for binary classification tasks, especially for imbalanced datasets, providing a more holistic assessment of prediction quality [24]

Future research should focus on expanding datasets to ensure broader geographic, demographic, and clinical variability. This would enhance the generalizability of findings and reduce biases related to unrepresentative datasets. Ground truth labeling processes should incorporate blinded evaluations and include inter-rater reliability metrics to improve the reliability of the Reference Standard. Models should undergo external validation using independent, real-world datasets to ensure robustness and clinical applicability. Additionally, providing detailed descriptions of dataset characteristics, patient selection criteria, and timing protocols is crucial to enhance transparency, reproducibility, and comparability across studies.

### Evaluation Metrics

The AI methods reviewed in this study employ a variety of evaluation metrics to assess their performance across different functionalities in chronic wound diagnosis. Most of these metrics are derived from the confusion matrix, which categorizes model predictions into true positives (TP), true negatives (TN), false positives (FP), and false negatives (FN) [23]. These metrics are summarized in Table 1.

Among these metrics, the AUC (Area Under the Curve) and the Receiver Operating Characteristic (ROC) curve are particularly significant. The ROC curve visually represents the trade-off between sensitivity (True Positive Rate) and the False Positive Rate (1-specificity) across various classification thresholds. It is used to evaluate the model’s ability to distinguish between classes. A perfect model achieves a curve that passes through the top-left corner (True Positive Rate = 1, False Positive Rate = 0), while a random classifier produces a diagonal line. The AUC is a scalar summary of the ROC curve, quantifying the overall discriminative performance of the model. It is particularly beneficial for imbalanced datasets, as it evaluates the model’s performance across all thresholds rather than relying on a single threshold. The AUC is directly related to the confusion matrix because it reflects the balance between true positives, false positives, true negatives, and false negatives. However, it does not depend on a fixed decision threshold, making it more comprehensive [25].

While these metrics are traditionally used in binary classification tasks, they are also extended to assess functionalities involving numeric outputs or multi-class tasks, such as wound area measurement and image segmentation. For example, wound area measurement uses regression-based metrics like Mean Absolute Error (MAE) and Root Mean Squared Error (RMSE) to quantify the accuracy of predicted wound areas relative to ground truth measurements. For image segmentation, metrics such as the Dice Similarity Coefficient (DSC) and Intersection over Union (IoU) are employed to evaluate the overlap between predicted and actual segmented wound areas. These metrics provide a nuanced evaluation of segmentation algorithms, which is critical for tasks requiring precise delineation of wound boundaries [26].

**Table 2:** Summary of articles with tissue classification implemented

Article	Technology	Validation methods	Wound type	Samples	Outcomes
Mukherjee et al. [28]	SVM, BN (RGB to HSI, fuzzy divergence-based thresholding)	Kappa Statistic for tissue pixel classification, Accuracy computation	Burn, DFU, Malignant ulcer, Pyoderma gangrenosum, VU, PU	74 images from Medetec medical image database	Accuracy: Granulation 86.94%, Slough 90.47%, Necrotic 75.53%
Ramachandram et al. [29]	Encoder-decoder CNN with EfficientNetB0	IoU, F1-Score, Precision, Recall	PU, AU, VU	58 images from Swift Medical’s Wound Database	Mean IoU = 0.8644 for wound segmentation, 0.7192 for tissue segmentation. High F1-scores for slough (0.731) and eschar (0.802)
Zahia et al. [30]	CNN (9 layers, 3 convolution)	Accuracy, Dice Similarity Coefficient, Sensitivity, Specificity	PU	22 images acquired from the Igurko Hospital, Bilbao-Spain	Accuracy 92.01%, Weighted DSC 91.38%, Precision for granulation 97.31%, necrotic tissue 96.59%, slough 77.90%
Veredas et al. [31]	K-means for segmentation, NN, RF, SVM for classification	Accuracy, Cohen’s kappa coefficient	PU	255 samples taken by clinicians from home-care patients	Accuracy: NN = 81.87%, RF = 87.37%, SVM = 88.08%
García-Zapirain et al. [32]	DeepMedic multi-pathway 3D CNN, Gaussian kernel smoothing	AUC, DSC, PAD	PU	193 images from healthcare services company in the Basque Country (Spain)	AUC = 95%, DSC = 92%, PAD = 10%

**Table 3:** Summary of articles with wound measurement implemented

Article	Technology	Validation methods	Wound type	Samples	Outcomes
Mohammed et al. [9]	AI-based wound assessment application (Swift Medical)	Intraclass correlation coefficient (ICC) test	VU, DFU, SW	91 patients with 115 wounds	AI tool was significantly faster with an average time of 62 s per assessment
Chan et al. [33]	CARES4WOUNDS (C4W) system	Intra- and inter-rater reliability via intraclass correlation statistics	DFU	75 wound episodes from 28 patients (547 images)	Intra-rater reliability ranged 0.933$-$0.994; inter-rater reliability for length = 0.947
Ferreira et al. [35]	Computer vision (Grayscale, Canny detection, OpenCV)	Mean absolute error (MAE)	CW	10 images from Science Photo Library, iStockPhoto and professionals	Higher error in desktop method than mobile device implementation
Foltynski et al. [36]	U-Net CNN for segmentation	Dice similarity, IoU, accuracy, specificity	DFU	565 samples captured with AreaMe software	Dice = 90.9%, IoU = 83.9%, accuracy = 99.3%, specificity = 99.6%; some errors on irregular edges
Niri et al. [37]	DL-based segmentation with 3D model reconstruction	Dice similarity, IoU, RMSE, MAE	DFU	569 CW, 270 DFU images from 7 patients	DICE increased from 36.53% to 86.3%, IoU from 29.48% to 77.09%, overall DICE = 93.04%
Simpson et al. [34]	Digital planimetry (ellipse, free hand ROI)	Intraclass correlation coefficient, mean, intra- and inter-rater reliability	Digital ulcers (SSc)	36 patients, 107 finger lesions	High reliability; uses calibrator to detect pixels and compute distances

In addition to these standard performance metrics, Intra- and inter-rater reliability are crucial for ensuring the quality of ground truth data in AI models. Intra-rater reliability measures the consistency of a single rater’s judgments over time, while inter-rater reliability evaluates agreement between multiple raters. Both are essential for validating datasets, particularly for subjective tasks like wound segmentation or classification. High reliability ensures consistent and unbiased annotations, reducing noise in training data and improving AI model performance. These metrics are typically quantified using tools such as the Intraclass Correlation Coefficient (ICC), which measures agreement in continuous data like wound area or volume and Cohen’s Kappa or Fleiss’ Kappa, which evaluate categorical agreement, such as wound classification into tissue types [27].

By employing these diverse metrics, the AI models reviewed in this study can be evaluated comprehensively across a wide range of chronic wound diagnosis tasks.

### Wound Tissue Classification

Wound tissue classification tasks focus on identifying different types of tissues within wounds (e.g., necrotic, granulation) using machine learning and deep learning approaches, such as Convolutional Neural Networks (CNNs) and Support Vector Machines (SVMs) (Table 2).

Mukherjee et al. explored the use of color and textural features combined with ML algorithms to classify granulation, necrotic, and slough tissues in chronic wounds. The images were segmented using fuzzy divergence-based thresholding, and pixel prediction was validated through statistical methods such as Kappa statistics. The results demonstrated that a SVM with a third-order polynomial kernel achieved high accuracy rates for classifying different tissue types, with accuracies of 86.94% for granulation, 90.47% for slough, and 75.53% for necrotic tissue. [28]. Similarly, the research from Veredas et al. used a CV approach combined with ML models to classify tissue types in wounds. By applying the k-means clustering algorithm for image segmentation and comparing several ML models, including neural networks (NN), SVM, and random forests (RF), the study achieved high performance rates. The accuracy rates varied depending on the model, with NN achieving 81.87%, RF at 87.37%, and SVM at 88.08% [31].

Ramachandram et al. employed a DL approach to automatically segment four key tissue types in chronic wounds: epithelial, granulation, slough, and eschar. Using an encoder-decoder model based on EfficientNetB0 architecture, the model was trained on a large dataset of 17,000 anonymized wound images. The segmentation model achieved high intersection-over-union (IoU) scores for wound segmentation (0.8644) and tissue segmentation (0.7192). While the model performed well in detecting slough and eschar (F1-scores of 0.731 and 0.802, respectively), it struggled with epithelial tissue, achieving a low precision and recall, resulting in an F1-score of 0.253 [29]. Zahia et al. report focused on pressure injuries, utilizing a nine-layer CNN for the segmentation of granulation, slough, and necrotic tissues. The model achieved a high overall accuracy of 92.01%, with a weighted Dice Similarity Coefficient (DSC) of 91.38%. The precision per class was particularly high for granulation tissue (97.31%) and necrotic tissue (96.59%), though slough tissue had a lower precision (77.90%) [30].

Using more advanced technology, Begoña et al. developed a multi-pathway 3D CNN called DeepMedic to segment and classify tissue types in pressure ulcers, including slough, granulation, and necrotic eschar. The model achieved strong performance metrics, with an average Area Under the Curve (AUC) of 95%, a DSC of 92%, and a Percentage Area Distance (PAD) of 10% [32].

**Table 4:** Summary of articles with wound segmentation and DL methods implemented

Article	Technology	Validation methods	Wound type	Samples	Outcomes
Ohura et al. [38]	Comparison of CNN architectures for segmentation (SegNet, LinkNet, U-Net, U-Net VGG16)	AUC, F1-score, Sensitivity, Specificity, Accuracy	PU, DFU, VLU	440 images (400 PU, 20 DFU, 20 VLU) from Kyorin University Hospital patients	The highest values obtained with UNet VGG16 (AUC 0.998). UNet faster than UNet-VGG16
Scebba et al. [39]	DL model compared U-Net, ConvNet, DeepLab, FCN for wound detection and segmentation	MCC, DSC, IoU	DFU, Digital Ulcers	1096 images from 76 patients	U-Net and FCN models MCC=[0.84$-$0.78] and MCC=[0.770$-$0.768], respectively
Wang et al. [41]	ResNet-based image recognition for diabetic foot wounds	Accuracy	DFU	727 images from Taichung Rongmin General Hospital	ResNet101_Kitti the highest precision (mAP of 87). ResNet101_fgvc the fastest (speed of 395ms)
Huang et al. [40]	MobileNetV2-based model compared to FCN-VGG16, SegNet, Mask-RCNN, U-Net	Precision, Recall, Dice Coefficient	DFU	1109 images from 889 patients	Recall 89.97%, Precision 91.01%, accuracy (DSC 90.47%), surpassing U-Net, Mask-RCNN and VGG16

**Table 5:** Summary of articles with wound segmentation and novel methods implemented

Article	Technology	Validation methods	Wound type	Samples	Outcomes
Dhane et al. [43]	MobileNet with location-enhanced convolution kernels for segmentation	IoU, Precision	CW	950 images from Medetec database	mean IoU 86.4680%, max. IoU 86.7427%, Precision = 95.0262%
Li et al. [42]	Spectral clustering with fuzzy similarity measures for segmentation	Accuracy, Sensitivity, Specificity, Dice Coefficient, JI	CW	70 images from 64 patients	The Db color channel 91.5% accuracy, 86.7% DSC, 79.0% JI, 87.3% sensitivity and 95.7% specificity
Gholami et al. [44]	Comparison of three edge-based algorithms for wound segmentation	Precision, Sensitivity, Specificity, JI, DS, HD	DFU, Burns, Scar	26 images from 15 subjects	Livewire best performance: 97.08%, 99.68% 96.67%, 96.22, 98.15, and 32.26, mean values, respectively, for accuracy, sensitivity, specificity, JI, DS, and HD
Wang et al. [45]	Wound boundary detection using associative hierarchical random field (AHRF)	Specificity, Sensitivity	DFU	100 images from 15 patients	specificity:>95% and sensitivity:>77%
Silva et al. [46]	Superpixel strategy using SVM and GrabCut	Accuracy, Precision, Specificity, Sensitivity, IoU	PU	105 images from MEDETEC	accuracy 96%, sensitivity 94%, specificity 97%, intersection over union 89% and precision 94%

**Table 6:** Summary of articles with wound segmentation and hybrid methods implemented

Article	Technology	Validation methods	Wound type	Samples	Outcomes
Heras-Tang et al. [47]	Five classifiers (DC, DT, GNB, LR, SVM), post-processing with DBSCAN clustering and morphological operators	Accuracy, Recall, Precision, F1 Score, AUC, JI	DFU	37 images from 30 Cuban patients	LR the best (JI 0.81, accuracy 0.94, recall 0.86, precision 0.91, and F1 score 0.88)
Curti et al. [48]	U-Net CNN model with transfer learning	DSC, Precision, Recall	CW	1564 images from 474 patients	Stable DSC (> 0.95) after 50 epochs

### Wound Measurement

Techniques of wound measurement aim to quantify wound characteristics like area, depth, and volume. Methods typically involve image processing models, including Mask R-CNN, to automate the measurement process, ensuring accuracy compared to manual assessments (Table 3).

Mohammed et al. compared the time efficiency of an AI-based wound assessment tool (Swift Medical) with manual methods in 91 patients with 115 wounds, including VU, DFU and SW. The results showed that the AI tool significantly reduced the time required for wound assessment, completing tasks in an average of 62 s. While the specific algorithms behind the tool were not detailed, the results suggest that AI-based wound assessment tools can streamline clinical workflows [9]. Similarly, in the study of Chan et al., the CARES4WOUNDS (C4W) system, an AI-enabled mobile application for wound imaging, was validated for diabetic foot ulcers. The study evaluated the intra- and inter-rater reliability of the tool by comparing its measurements with traditional manual methods across 547 images from 28 patients. The C4W system demonstrated high reliability, with intra-rater reliability ranging from 0.933 to 0.994 and inter-rater reliability for wound length measurements at 0.947 [33]. Simpson et al. measured digital ulcers in systemic sclerosis (SSc) using two digital planimetry methods: ellipse and free-hand Region of interest (ROI). The study, which involved 107 finger lesions from 36 patients, reported high intra- and inter-rater reliability. However, it relied on manual segmentation, which could introduce variability. The method involved using a calibrator to detect pixels and compute distances but did not fully explain how the measurement was performed [34].

Ferreira et al. implemented basic CV techniques such as grayscale conversion, Canny detection, and OpenCV for wound area measurement in 10 images. This method showed higher error rates when implemented on a desktop machine compared to a mobile device. The study highlighted technical challenges such as metadata loss, indicating that more advanced technology is needed for accurate and functional wound area measurement [35].

Foltynski et al. implemented an internet-based service for automatic wound area measurement using U-Net CNN applied to DFU. The system achieved high performance, with a DSC of 90.9%, an IoU of 83.9%, an accuracy of 99.3%, and specificity of 99.6%. However, the system struggled with detecting wound margins in cases of irregular edges, which suggests room for improvement in edge detection for chronic wounds [36]. Lastly, the Niri et al. study aimed to improve wound segmentation using a DL-based method with multi-view data augmentation and 3D surface modeling. Tested on 569 CW and 270 DFU images, the results showed significant improvements in DSC and IoU scores, rising from 36.53% to 86.3% and 29.48% to 77.09%, respectively. The study concluded that the 3D modeling approach significantly improved segmentation performance, achieving an overall DSC of 93.04% [37].

### Wound Segmentation

Segmentation methods use algorithms to identify and outline wound boundaries within an image. Models such as U-Net and Mask R-CNN are commonly employed to isolate wound regions for further analysis or measurement.

Several articles focus on wound localization and segmentation as a primary step for automating wound care. Various CNN architectures were compared in the context of wound segmentation (Table 4). Ohura et al. demonstrated that U-Net with a VGG16 encoder pre-trained on ImageNet produced the best results for DFU and VU, with an AUC of 0.997, specificity of 0.943, and sensitivity of 0.993. U-Net was noted for its practical application due to faster segmentation speeds compared to other architectures such as SegNet and LinkNet [38]. Also, in a comparative study [39] of DL-based segmentation methods, including U-Net, ConvNet, DeepLab, and Fully Convolutional Networks (FCN), the U-Net model performed well across varying training set sizes, maintaining segmentation performance with a Matthews correlation coefficient (MCC) in the range of 0.768$-$0.84 even with reductions in the training set size. Similarly, [40] demonstrated that Mask-RCNN achieved the highest precision in chronic wound segmentation, with 94.3% precision and a recall of 86.4%. In contrast, U-Net showed superior recall, achieving 91.29%, and slightly lower precision of 91.01%. Huang et al. applied three deep neural networks, including Fast R-CNN ResNet101 and Inception, to identify and refine wound boundaries using GrabCut and SURF algorithms. Among the trained models, ResNet101_Kitti had the highest precision (mAP of 87), while ResNet101_fgvc was the fastest (speed of 395ms) [41].

Novel techniques such as fuzzy spectral clustering were applied to wound region delineation and achieved an accuracy of 91.5% with a DSC of 86.7% on a set of 70 chronic wound images [42]. Another innovative approach [43] involved a wound segmentation network that enhanced location information, yielding a mean IoU of 86.47% and a precision of 95.03% from 950 images. Work from Gholami et al. compared three edge-based algorithms for chronic wound segmentation found that the Livewire (Intelligent Scissors) technique outperformed others, achieving mean values of 97.08% accuracy, 99.68% sensitivity, and 96.67% specificity across 26 images from 15 subjects, along with excellent Jaccard index and DSC scores [44]. In another study [45], the automatic measurement of PU using SVM and the GrabCut method showed an accuracy of 96%, sensitivity of 94%, and a specificity of 97% across 105 images from the public MEDETEC database. Similarly, a framework [46] using associative hierarchical random field (AHRF) for foot ulcer detection achieved specificity rates above 95% and sensitivity above 77% in tracking 15 patients over two years. Novel techniques of wound measurements are summarized in Table 5.

**Table 7:** Summary of articles with wound segmentation and ML methods implemented

Article	Technology	Validation methods	Wound type	Samples	Outcomes
Yadav et al. [49]	k-means and fuzzy c-means clustering using YDbDr color space	Accuracy, PPV, Sensitivity	PU, DFU, VU, MU, PG	77 images from Medetec database	Most accurate in the Db channel (accuracy fuzzy c-means 84.20% and k-means 82.39%. Accuracies 62.29% and 66.20% in other channels
Atisattapong et al. [50]	PSO for binary image segmentation compared with Otsu method	Visual validation	PU, VU, AU, Burns, Scalds	50 images from Medetec database	46% of cases improve segmentation, 16% unchanged quality and 38% quality degraded
Dhane et al. [51]	Spectral clustering for wound bed identification	Accuracy, Sensitivity, PPV	PU, LU, DFU	105 images from 64 participants	Accuracy 86.73%, with 91.80% PPV and 89.54% sensitivity; k-means the lowest accuracy; highest sensitivity with fuzzy c-means (87.98% and 88.77%)
Lu et al. [52]	CNN model fwith color correction	Accuracy	CW	300 images from Medetec database	Average accuracy rate improved about 3% than the traditional methods
Veredas et al. [53]	K-means based statistical color models	AUC, F-Score, Kappa, Sensitivity, Specificity, Accuracy	PU	435 images from 69 patients	AUC 94,26% (SD.0563); accuracy: 87,77% (SD.0799); F-score 73,89% (SD.1550); Cohen’s kappa 65,85% (SD.1787)

**Table 8:** Summary of articles with real-time CW detection on mobile devices

Article	Technology	Validation methods	Wound type	Samples	Outcomes
Goyal et al. [54]	Deep learning models (MobileNet, Inception-V2)	Speed, Model Size, mAP, Overlap Percentage	DFU	1775 images from the Lancashire Teaching Hospitals	Averaged precision of 91.8%, InceptionV2 the best performance. SSD-Mobilenet faster model but worst in accuracy
Wagh et al. [55]	AHRF vs. CNNs (FCN, U-Net, DeepLabV3)	Dice Score, Inference Time	CW	1442 images from local capture, scraping internet and from University of Massachusetts Medical Center	AHRF outperforms U-Net on small datasets (< 300 images) but is slower and less accurate than FCN and DeepLabV3, while CNNs are superior on larger datasets
Anisuzzaman et al. [56]	2D wound images and YOLOv3 vs. SSD	Precision, Recall, F1-score, IoU, mAP	DFU, PU, VU	1800 images (from AZH and MEDETEC)	mAP 0.97; Superior to SSD with a 7.5% higher mAP on AZH dataset

**Table 9:** Summary of articles on wound classification using variants of CNN model

Article	Technology	Validation methods	Wound type	Samples	Outcomes
[57]	DFUNet, CNN-based model	Sensitivity, specificity, F-measure, AUC	DFU	292 images from Lancashire Teaching Hospitals	93.4% sensitivity, 0.962 AUC
[58]	DFU-QUTNet CNN, SVM	Precision, recall, F1-score	DFU	754 images from the Nasiriyah Hospital’s diabetic center	DFU-QUTNet with SVM 95.4% precision and 94.5% F1-score
[59]	Faster R-CNN, deep learning	Accuracy, sensitivity, MCC, AUC	DFU	1249 ischemia, 628 infection cases from the Lancashire Teaching Hospitals	accuracy ischemia 90%, infection 73%
[60]	Class Knowledge Bank (CKB), ResNet, EfficientNet, and DeiT	Accuracy, sensitivity, precision, F1-score, AUC	DFU	628 infection, 1249 ischemia cases from the Lancashire Teaching Hospitals	CKB-DeiT-B-D achieved F1-score of 78.20 and AUC of 84.78
[61]	Multi-modal classifier (VGG16, ResNet50)	Accuracy, precision, recall, F1-score	DFU, PU, VU, SW	730 images (AZH), 358 (Medetec)	Highest accuracy of 86.67% on Medetec dataset using VGG19 + MLP
[62]	CNN architectures (AlexNet, VGG16, ResNet50, etc.)	Sensitivity, specificity, accuracy, MCC	DFU	1459 images from DFU2020 database	ResNet50 accuracy ischemia 99.49%, infection 84.76%
[63]	CNN-GLCMNet combining CNN and GLCM	Recall, specificity, precision, accuracy	DFU	756 images from the diabetic center of Nasiriyah’s Hospital	DNN 97.43% accuracy with healthy and infected
[64]	DFINET, CNN-based model	Accuracy, MCC	DFU	5890 images from the Lancashire Teaching Hospitals	91.98% accuracy for infection classification
[65]	Mask-RCNN for segmentation and classification of PU stages 1-4	Precision, Recall, Accuracy, F1 Score	PU	969 images provided by eKare Inc. (Fairfax, VA)	Mask-RCNN Acc. 92.6% classification and 93.0% segmentation. F1-scores [0.842$-$0.947]
[66]	ResNet, CNN (Res4Net, Res7Net)	Accuracy, F1-Score, AUC, MCC	DFU	210 ischemia, 628 infection cases from Department of Computing and Mathematics	Res4Net accuracy ischemia 97.8%, Res7Net AUC infection 0.889

**Table 10:** Summary of articles on wound classification using hybrid CNN models

Article	Technology	Validation methods	Wound type	Samples	Outcomes
[67]	Hybrid CNN models	Precision, recall, F1-score	DFU	754 images from the Nasiriyah Hospital’s diabetic center	Achieved 97.3% precision, 94.5% recall, and 95.8% F1-score
[68]	Fusion of Gabor, HOG, and deep features	AUC, sensitivity, precision, accuracy	DFU	1679 images (healthy), 9870 (ischemic), 5892 (infected)	RF classifier significantly increased AUC to 0.97 (ischemia) and 0.81 (infection)
[69]	Ensemble CNN classifier (AlexNet)	Accuracy, precision, recall, F1-score	DFU, VU, SW	400 images from the AZH Wound and Vascular Center in Milwaukee, Wisconsin	94.28% average accuracy for binary classification, 91.9% for multiclass
[70]	EfficientNet	Sensitivity, precision, FPR, AUC	DFU	3000 images from The Diabetic Foot Ulcers Grand Challenge (DFUC) 2021	99% accuracy for ischemia and 98% for infection

Other studies incorporated hybrid techniques to improve segmentation accuracy (Table 6). For example, Heras-Tang et al. developed a method combining logistic regression (LR), DBSCAN clustering, and morphological operations for DFU segmentation achieved the best Jaccard index of 0.81, accuracy of 94%, and F1 score of 0.88 with the LR model on a dataset of 140 images [47]. Another study [48] using semi-supervised active learning and transfer learning demonstrated high efficiency in wound segmentation tasks. After fewer than 50 training epochs, a CNN-based architecture pre-trained on ImageNet achieved a stable DSC of 0.95, showing effective performance on a dataset of over 1,500 chronic wound images.

Additionally, color space selection played a crucial role in segmentation accuracy in several articles (Table 7). With the YDbDr color space, Yadav et al. obtained the highest contrast and accuracy for wound and non-wound region segmentation using k-means and fuzzy c-means clustering [49]. Atisattapong et al. assessed the use of Particle Swarm Optimization (PSO) combined with binary image segmentation to optimize thresholding for chronic wound assessment, achieving improved segmentation in 46% of cases when compared to the traditional Otsu method, though it provided limited insights overall [50]. Dhane et al. utilized spectral clustering for unsupervised segmentation of lower extremity wound beds, demonstrating a segmentation accuracy of 86.73%, with positive predictive values of 91.80% and sensitivity of 89.54%, surpassing k-means and fuzzy c-means methods [51]. Lu et al. introduced a CNN-based method that included a fast level set model for intensity correction and color adjustments, reporting a 3% improvement in average accuracy over traditional methods when tested on 300 chronic wound images [52]. Lastly, an approach [53] employing statistical color models for wound area detection achieved a high AUC of 0.9426 and accuracy of 87.77% across 435 images from home-care patients.

Finally, approaches have been identified for real-time CW detection on mobile devices (Table 8). A report [54] using MobileNet and Inception-V2 models demonstrated varying performance in terms of speed and accuracy. MobileNet was superior in terms of speed and model size, but Faster R-CNN with Inception-V2 showed better accuracy in localizing DFU, achieving an average precision of 91.8% and indicating that real-time performance considerations may require balancing accuracy with processing efficiency. Wagh et al. assesses the performance of Associative Hierarchical Random Fields (AHRF) against three DL models (FCN, U-Net, and DeepLabV3) across various datasets. The findings indicate trends in performance based on dataset size and model type, showing how AHRF compares to DL models in terms of accuracy and speed for wound image segmentation tasks [55]. Finally, a mobile app developed for wound localization using the YOLOv3 deep neural network demonstrated a high performance with a mean Average Precision (mAP) score of 0.97. The YOLOv3 outperformed the SSD model by 7.5% on the AZH dataset consisting of 1,800 images from DFU, PU AND VU [56].

### Wound Classification

These techniques classify wounds based on their type (e.g., diabetic, venous, pressure ulcers) by analyzing wound features through ML like SVM or RF. They help in understanding wound etiology and tailoring treatment plans.

Several models have been developed to improve wound classification, particularly for diabetic foot DFU, PU, and other chronic wounds (Table 9). Goyal et al. introduced a CNN to distinguish between healthy and DFU-affected skin. It compared DFUNet to LeNet, AlexNet, and GoogLeNet, achieving a sensitivity of 93.4% and an AUC of 0.962 [57]. Another report [58] proposed a network architecture that increased network width while maintaining depth for effective DFU classification. The model, trained on 754 DFU images, achieved 95.4% precision, 93.6% recall, and 94.5% F1-score, showcasing its robustness. An article [59] focused on ischemia and infection classification in DFUs, comparing models like InceptionV3, ResNet50, and InceptionResNetV2. Ensemble CNN models achieved an accuracy of 90% for ischemia and 73% for infection, outperforming traditional ML algorithms. This concept was further expanded by Xu et al., which introduced a Class Knowledge Bank (CKB) approach leveraging models like ResNet, EfficientNet, and DeiT to classify DFU infection and ischemia. The CKB-DeiT-B-D model achieved an F1-score of 78.20% and an AUC of 84.78%, surpassing other models in performance [60]. Beyond DFU classification, Anisuzzaman et al. combined wound images and location information using VGG16, ResNet50, and LSTM models to classify multiple wound types including DFUs, PUs, VUs, and SWs. This approach attained the highest accuracy of 86.67% on the Medetec dataset when using a VGG19 + MLP combination [61]. Ahsan et al. employed architectures such as AlexNet, VGG16, and ResNet50 on the DFU2020 dataset, achieving a maximum accuracy of 99.49% for ischemia and 84.76% for infection classification with ResNet50 [62]. In another study [63], introduced the CNN_GLCMNet model, which combined GLCM features with deep learning, achieving 97.43% accuracy on a dataset of 756 images. Protik et al. presented DFINET, a 22-layer CNN that attained 91.98% accuracy on 5,890 images, demonstrating its effectiveness in infection detection [64]. In addition to segmentation, a study [65] integrated wound classification techniques using Mask-RCNN for the classification of PUs in different stages showed an overall classification accuracy of 92.6% across 969 images, with F1 scores for stages 1-4 of PUs ranging from 0.842 to 0.944. Additionally, Das et al. utilized ResNet for classifying ischemia and infection, achieving an impressive AUC of 0.9968 for ischemia detection [66].

The exploration of hybrid models further enhanced classification accuracy in wounds (Table 10). Alzubaidi et al. assessed four hybrid CNN models, achieving a precision of 97.3% and a recall of 94.5% on 754 images [67]. Al-Garaawi et al. introduced a fusion-based approach that combined hand-crafted features like Gabor and Histogram of Oriented Gradients (HOG) with deep features extracted from a GoogleNet CNN. This approach improved AUC scores to 0.97 for ischemia detection and 0.81 for infection classification [68]. The report [69] utilized an ensemble CNN (AlexNet) for categorizing wound images, achieving an average classification accuracy of 94.28% for binary and 91.9% for multiclass problems across 400 images. Furthermore, Liu et al. utilized EfficientNet, which achieved 99% accuracy for ischemia and 98% for infection classification, showcasing the effectiveness of this architecture [70].

Several studies applied traditional ML techniques to address wound classification (Table 11). Hu et al. used decision trees (DT), LR, and RF, with RF demonstrating an AUC of 0.864 across 11,838 records [71]. Sotoodeh et al. applied LR, RF and NN to detect and classify PU based on nurse notes in the MIMIC-III dataset. The analysis on of 3,589 cases used the Scispacy tool for named entity recognition and NegEx for negation detection. RF achieved the highest AUC (95%), making it the best-performing model for interpreting clinical notes related to pressure ulcers [72]. In Moon et al. study, DT analysis revealed significant risk factors for PU development, achieving an accuracy of 80.4% [73]. Additionally, Silva et al. employed a K-means clustering approach to classificate patients into high- and low-risk groups based on self-care behaviors, foot care habits, and social conditions, and achieving 97% accuracy in identifying high-risk patients based on a dataset of 153 individuals [74].

**Table 11:** Summary of articles on wound classification using ML models

Article	Technology	Validation methods	Wound type	Samples	Outcomes
[71]	DT, LR, RF	Precision, recall, specificity, F1-score, AUC	PU	11838 inpatient records	RF achieved best performance with AUC of 0.864
[72]	LR, RF, NN	AUC, ROC, F1 score	PU	3589 cases (MIMIC-III dataset)	RF achieved 95% AUC for PU detection
[73]	Decision tree analysis	Accuracy, sensitivity, specificity	PU	15856 cases from the 2014 NIS provided by HIRA (HIRA-NIS-2014-0071)	80.4% accuracy, identifying length of stay and comorbidity as key factors
[74]	K-means clustering	Silhouette, accuracy	DFU	153 patients	97% accuracy for high-risk DFU classification

**Table 12:** Summary of articles developing specialized models for specific wound classifications

Article	Technology	Validation methods	Wound type	Samples	Outcomes
[75]	CNN model	Accuracy, sensitivity, specificity, AUC	Deep wound, infected wound, AU, VU, PU	2149 images from the Chang Bing Show Chwan Memorial Hospital, Changhua, Taiwan	96% accuracy for venous ulcer classification
[76]	FusionSegNet, CNN, MobileNetV2, Residual U-Net	AUC, accuracy, sensitivity, specificity, F1-score	DFU	1211 images from Shanghai Municipal Eighth People’s Hospital	95.78% accuracy, 94.27% sensitivity, and 96.88% specificity
[77]	XAI techniques, VGG16 CNN	Precision, recall, F1-score, AUC	DFU, lymphovascular injury, SW, PU	8690 images from the eKare Inc	DFU precision of 0.85 and F1-score of 0.92
[78]	Image identification algorithm	Judgment results of expert physicians	PU	50 patients	82.4% sensitivity and 100% specificity for necrosis detection
[79]	DCNN, transfer learning	Recall, precision, F1-score	DFU	1200 images from different domains	97.6% F1-score using domain-specific transfer learning

**Table 13:** Summary of articles on wound classification using YOLO-based models

Article	Technology	Validation methods	Wound type	Samples	Outcomes
[80]	YOLOv5 object detection model	Precision, recall, mAP	PU	1000+ images from Medetec image database	76.9% mAP for PU classification
[81]	CNN and YOLOv2	Accuracy, sensitivity, confidence scores	DFU	1249 ischemia, 831 infection cases from DFU-Part(B) dataset	99% accuracy for infection and ischemia classification, 0.973 confidence score for localization
[82]	YOLOv5 models	mAP, IoU	VU, DFU	885 images from the Christian Hospital Melle and from the University Hospital Essen	YOLOv5m6 model achieved highest precision (0.942) and recall (0.837)

Specialized models have been developed to target specific classifications within wound management (Table 12). Huang et al. presented a CNN model that classified multiple wound types, achieving 96% accuracy in the venous ulcer classification task using 2,149 images [75]. The study [76] integrated global foot features with local wound features, achieving 95.78% accuracy on 1,211 DFU images. Explainable AI techniques have also been applied in chronic wound classification. Sarp et al. utilized XAI tools on a dataset of 8,690 images, achieving a precision of 0.85 and an F1-score of 0.92 for DFU classification [77]. Sakakibara et al. developed an image-based algorithm that detected necrosis in PUs by analyzing color pixels and luminance differences. The algorithm achieved a sensitivity of 82.4% and specificity of 100% in identifying necrosis and its type (black or white), emphasizing the importance of specialized algorithms for wound-specific tasks [78]. Moreover, Alzubaidi et al. explored transfer learning in DFU classification, achieving a remarkable F1-score of 97.6% with a dataset of 1,200 images, emphasizing the effectiveness of domain-specific approaches [79].

Several studies harnessed YOLO-based models for effective wound detection and classification (Table 13). One article [80] achieved a mAP of 76.9% on over 1,000 images, showcasing its efficacy in detecting pressure ulcers. Amin et al. combined CNN for classification and YOLOv2-DFU for localization, achieving 99% accuracy in a dataset comprising ischemia and infection cases [81]. In addition, Husers et al. employed YOLOv5 models to classify DFU and VU, with the YOLOv5m6 model achieving a precision of 0.942 and recall of 0.837 on 885 images [82].

Further contributions in the field include a report [83] which compared a modified ResNet with SVM, RF, and Gradient Boosted Decision Trees (GBDT), achieving an AUC of 83.3% with ResNet, outperforming SVM (44.4%), random forest (67.1%), and gradient boosting classifier (66.9%) for wound infection detection on a dataset of 480 images. Another article [84] explored factorization-based segmentation for pressure and venous ulcers using MLP, SVM, RF, and NB, with MLP achieving the highest accuracy (83.1%) compared to SVM (79.7%), RF (79.7%), and Naïve Bayes (72.9%). In [85] utilized transfer learning from InceptionV3, ResNet50, and VGG16 to classify ulcers, achieving a sensitivity of 97% with VGG16, significantly outperforming dermatologists (sensitivity 72.7% for experts, 45.5% for juniors). Additionally, the study [86] compared SVM, DT, RF, and artificial neural networks (ANN) for predicting pressure ulcers, finding that RF achieved the highest accuracy and AUC values (above 0.95). Sin et al. applied LR, KNN, SVM, RF, MLP, and BN, with RF performing best with 96% accuracy based on 4,652 patient records [87]. Also, [88] demonstrated a residual network variant achieving 98.79% accuracy in classifying oral ulcer images. Finally, Reddy et al. proposed the use of Extreme Learning Machine (ELM) for DFU detection, which achieved 96.15% accuracy, outperforming KNN, SVM, and ANN [89]. Additionally, Pereira et al. implemented a DL segmentation model, achieving a mean IoU of 89.9% on 1,337 images [90]. The report of the articles are summarised in the Table 14.

**Table 14:** Summary of articles on wound classification with comparative studies of ML models

Article	Technology	Validation methods	Wound type	Samples	Outcomes
[83]	Expert system, ResNet-based model	Accuracy, recall, precision, F1-score, AUC	SW	480 wound photographs from 100 surgical patients	ResNet achieved 83.3% AUC for infection detection, outperforming other classifiers
[84]	MLP, SVM, RF, NB	Accuracy	PU, VU, AU	59 images from the Medetec Medical Image Database	MLP achieved highest accuracy (83.05%) for binary classification
[85]	InceptionV3, ResNet50, VGG16	Sensitivity, specificity, accuracy	Pyoderma gangrenosum, leg ulcers	491 images from patients treated in two large dermatology centers	CNN sensitivity (97%) outperformed dermatologists
[86]	SVM, DT, RF, ANN	Accuracy, AUC	PU	5814 patients	RF model achieved the highest accuracy and AUC (both > 0.95)
[87]	LR, KNN, SVM, RF, MLP, BN	AUC, ROC	PU	4652 patients	RF achieved 96% accuracy
[88]	Variant of Residual Network	Sensitivity, specificity, accuracy	Oral ulcers	360 images from dataset labeled by dental specialists from Fujian Stomatological Hospital	98.79% accuracy for oral ulcer classification
[89]	ELM, KNN, SVM, ANN	Accuracy, TS/CSI, FDR	DFU	22 attributes and 133 instances from the ”Figshare” data repository	ELM achieved 96.15% accuracy, outperforming KNN, SVM, and ANN
[90]	Deep learning segmentation, machine learning models	Accuracy, precision, recall, F1, AUC	SW	1337 images from 34 cardiothoracic surgery patients of Hospital de Santa Marta during a 30-day follow-up	87.6% recall for leg wounds

### Wound Healing

These methods predict wound healing progress or identify risks for delayed healing based on factors like tissue health or wound size. ML algorithms are used to assess the likelihood of healing or complications, aiding clinical decision-making. Notably, the evaluation of wound healing progress in chronic wounds often relies on the predictive scar factor, which is methodologically based on weekly assessments over 4 consecutive weeks. This approach is essential for establishing accurate healing metrics and for providing comparative parameters that allow AI tools to optimize their learning and prediction capabilities [91].

In the field of DL, several studies have explored the application of CNNs for wound assessment (Table 15). A Reduced ResNet-18 model was employed in [92] to detect granulation tissue in DFU, demonstrating an IoU rate above 0.5 for identifying tissue growth. Similarly, a DenseNet CNN framework with patch-based attention was used in [93] to assess multiple wound attributes, achieving accuracy and F1 scores above 0.8. Another study [94] introduced the Semi-Supervised PMG EfficientNet architecture, improving wound assessment accuracy to 90% by augmenting the WoundNet dataset with a Progressive Multi-Granularity mechanism. In self-care, a smartphone-based system allowed patients to assess surgical wounds by using a variety of classifiers, such as CART and Naïve Bayes, reaching over 90% accuracy in wound state evaluation [95]. In another study [96], smartphone and tablet cameras were used to capture DFU images for healing prediction, employing ResNet50 for feature extraction and RF and SVM for classification. The study demonstrated higher AUROC values (0.734) when combining all features and showed improved performance when using handcrafted imaging features (0.760$-$0.794) compared to clinical features alone. Also, a large-scale AI model, AutoTrace, for wound healing prognosis, was developed by Gupta et al.based on over 2 million wound evaluations, achieving an IoU score of 0.86 and an 8-13% improvement over tools like the PUSH and the BWAT. While PUSH is specific to pressure injuries and BWAT is broader, covering venous, diabetic, and arterial ulcers, both have limitations in addressing diverse wound types [16].

**Table 15:** Summary of articles on wound healing prediction with DL models implemented

Article	Technology	Validation methods	Wound type	Samples	Outcomes
[92]	Reduced ResNet-18 for granulation tissue detection	IoU	DFU	219 images from 100 patients	IoU rate higher than 0.5
[93]	DenseNet CNN framework with patch-based attention	Accuracy, F1, sensitivity, specificity	DFU, PU, VU, SW	1639 images from WoundNet database	F1 scores > 0.8
[94]	Semi-Supervised PMG EfficientNet, augmented WoundNet dataset	Accuracy, F1, Sensitivity, Specificity	DFU, PU, VU, SW	1639 labeled images from WoundNet dataset, 9870 unlabeled DFU images from DFUC 2021	classification accuracies and F1 around 90% in both
[95]	Various ML classifiers: CART, GNB, KNN, LR, DNN, RF, SVM	Accuracy, Precision, Recall, F-measure	SW	131 images from 46 patients	90% state assessment accuracy
[96]	Matlab for segmentation, ResNet50 for feature extraction, RF, SVM	Precision, Recall, F1, AUROC	DFU	208 wounds from 113 patients	AUROC 0.734$-$0.794 for hand-crafted features
[16]	AutoTrace deep learning model for wound segmentation and healing prediction	mIoU	PU, VU, DFU, AU	2,151,185 evaluations from 98,407 patients	5-13% improvement over Pressure Ulcer Scale for Healing (PUSH), the Bates-Jensen Wound Assessment Tool (BWAT). 0.86 mIOU for segmentation, tissue mIOUs from 0.42 to 1.0

**Table 16:** Summary of articles on wound healing prediction with ML and hybrid models implemented

Article	Technology	Validation methods	Wound type	Samples	Outcomes
[97]	Naïve Bayes classifiers for SF and BF, comparison with logistic regression	Accuracy, sensitivity, specificity, AUC	SW	851 participants	SF AUC 0.760 significantly better than BF (AUC 0.670)
[98]	SVM, NB, KNN, RF, GLM, and boosting to predict DFUs	Accuracy, Precision, Recall, AUC	DFU	362 patients	NB model AUC 0.864, recall 0.907, F1 0.744
[99]	PWAT, SMOTE, DT, SVM, RF, MLP, XGBoost	Precision, Recall, F1	DFU, VU, AU	205 images from a corpus of IRB-approved UMMS patient data	XGBoost accuracy 81% (both visual + textual)
[100]	LR, RF, GBDT, DNN, SHAP analysis for variables	AUC	CW	461,293 patients, 1,220,576 wounds	GBDT AUC 0.854$-$0.855
[14]	LR and RF for risk stratification using registry data	Accuracy, ROC	DFU	246,705 patients, 13,695 DFU, 7,540 amputations	socioeconomic and medical factors affect risk
[101]	LR, RF, ANN using OASIS and NLP for risk prediction	Sensitivity, Specificity, Accuracy, AUC	CW	112,789 patients	LR AUC 0.82, ANN PPV 3.8%
[102]	LASSO, RF, GBT models for delayed healing	AUC, Brier Reliability	CW	59,953 patients	GBT AUC 0.842

On the ML side, a variety of models were used to predict wound healing outcomes and risk factors (Table 16). Naïve Bayes (NB) classifiers combined with serial wound characteristics significantly improved the prediction of surgical site infections (SSI) compared to baseline risk factors [97]. Additionally, ML algorithms, such as SVM, NB, RF, were employed in predicting hard-to-heal DFUs [98], with the NB model achieving the best results (AUC 0.864). In another study [99], ML classifiers generated actionable decisions for chronic wound care, with XGBoost achieving 81% accuracy when combining visual and textual data. Other predictive models like LR, RF, and GBDT were used to forecast wound healing times in large datasets [100], yielding AUCs around 0.85 for 4-, 8-, and 12-week healing probabilities. Another study [14] utilized LR and RF classifiers for a risk stratification analysis of DFU and amputation, incorporating both socioeconomic and medical data from over 246,705 patients with diabetes. It identified significant risk factors such as cardiovascular disease, peripheral artery disease, and neuropathy. Additionally, the study reported an inverse correlation between disposable income and the risk of DFU and amputation. However, while the study integrates socioeconomic factors with medical data, the exact computational interaction between these variables and the AI models is not explicitly detailed.

Finally, hybrid approaches combining structured and unstructured data were used to enhance predictive accuracy (Table 16). For example, a study [101] on wound infection-related hospitalizations utilized LR, RF, and ANN, showing significant improvements in predictive performance when both structured OASIS-C data and unstructured clinical notes were integrated. Similarly, a GBDT model was developed for rapid identification of slow-healing wounds, achieving an AUC of 0.842 [102], highlighting the importance of wound characteristics and patient care status in predictive modeling.

### Multiple Methods

Several studies utilized CNN and related architectures to segment wound tissues and assess wound characteristics (Table 17). For example, Blanco et al. used a Reduced ResNet-18 model for identifying granulation tissue in DFUs, showing that IoU rates exceeded 0.5 across 219 images from 100 patients [103]. Similarly, Zahia et al. employed Mask R-CNN with ResNet backbones on a dataset of 210 wound images, achieving high DSC (0.83) and precision (0.87) for wound segmentation, with low errors in depth estimation [104]. The Comprehensive Wound Image Assessment by Chang et al. also applied DeepLabV3, reaching impressive accuracy for both tissue classification and wound segmentation (precision 0.9915) on over 2800 images [105]. Similarly, Chino et al. employed an encoder/decoder neural network to segment venous and AU across 446 images. The model achieved a DSC score above 90% for wound segmentation, outperforming QTDU and DeepLabv3+ by up to 16%. Additionally, it estimated the wound area with a relative error of 12.1% [106]. Other efforts also leveraged DenseNet CNN frameworks with patch-based attention, as demonstrated by Chakraborty et al., where the model successfully classified different wound attributes in 1639 images with F1 scores exceeding 0.8 for DFU, PU, VU and SW [107].

**Table 17:** Summary of articles on various methods with CNN approaches implemented

Article	Technology	Validation methods	Wound type	Samples	Outcomes
[103]	InceptionV3 and ResNet models with superpixel-driven segmentation for ulcers’ quality assessment	Cohen-Kappa, Coefficient, F1-Score, Sensitivity, Specificity, and AUC	AU and VU	217 photos from ULCER_SET database with images from Neurovascular Ulcers Outpatient Clinic of HCFMRP/USP	QTDU accurately spots wounded tissues (AUC = 0.986, sensitivity = 0.97, and specificity = 0.974) with an F1-score improvement up to 8.2% using a ResNet-based model
[104]	CNN for wound segmentation and 3D mesh for depth, volume, area, and axes computation (Mask R-CNN with ResNet50 and ResNet101)	Dice Similarity Coefficient (DSC), Sensitivity, Precision for segmentation, MAE, RMSE for measurement evaluation	PU	210 photos (110 from hospitals, 100 from Medetec MIOD)	Best segmentation results with Mask-RCNN (mean Dice score: 0.83, sensitivity: 0.85, precision: 0.87), MAE for wound depth: 0.74 cm, volume: 4.69 cm3
[105]	Region-based labeling method with DL models (U-Net, DeeplabV3, PsPNet, FPN, Mask R-CNN with ResNet-101) for segmentation	F1-score, IoU, Precision, Recall, Accuracy	PU	2836 images labeled for tissue classification, 2893 for re-ep segmentation	DeeplabV3 performed best with precision of 0.9915, recall of 0.9915, and accuracy of 0.9957 for tissue classification
[106]	Encoder/decoder DNN to segment wound area, detects ruler/tape for pixel density estimation	Dice Score, Jaccard Coefficient, Precision, Recall	VU and AU	446 images from ULCER and ULCER-2 database	Dice score greater than 90%, able to estimate wound area with a relative error of 12.1%
[107]	Fuzzy c-means clustering and machine learning for wound tissue classification	Accuracy compared with expert clinicians’ manual segmentation	CW	153 images (60 granulation, 20 slough, 53 necrosis)	93.75% overall accuracy, Random Forest provided 85.67% accuracy

Some studies integrated semi-supervised learning techniques or hybrid architectures to handle limited data availability or augment existing datasets (Table 18). Da Silva et al. proposed a semi-autonomous YOLO V2 CNN model to classify wound tissues using 1,194 images, although it achieved a relatively modest mAP of 21.32% [108]. On the other hand, Liu et al. compared U-Net and Mask R-CNN for PU segmentation, where U-Net achieved significantly higher segmentation accuracy (DSC of 0.8448) [109]. Furthermore, Rajathi et al. combined CNNs with active contour techniques to classify varicose ulcer tissues with remarkable accuracy (99.55%) [110]. Also, in [111] Mask R-CNN was validated using 330 images to segment wounds compared to expert clinicians’ assessments. It achieved higher processing speed, reproducibility, and interclass correlation coefficient (ICC) values of 0.77 for software-based analysis compared to 0.34 for ruler-based methods. Intra-rater reliability was excellent, with ICC values of 0.99.

**Table 18:** Summary of articles on various methods with semi-supervised learning techniques implemented

Article	Technology	Validation methods	Wound type	Samples	Outcomes
[108]	Semi-autonomous application using YOLO V2 CNN for tissue classification and treatment suggestions	Accuracy/recovery curve, Recall, Precision	CW	1194 photos from the private image base of the stomatherapist consulted with 1992 discriminated objects	General average precision for CNN of 21.32%
[109]	Mask R-CNN and U-Net trained for segmentation and automatic wound area measurement via LiDAR camera	DSC, IoU	PU	528 images of patients from National Taiwan University Hospital	U-Net outperformed Mask R-CNN in segmentation (Dice coefficient: U-Net = 0.8448, Mask R-CNN = 0.5006), 26.2% relative error for wound area measurement compared to manual methods
[110]	Active contour technique with gradient descent for wound segmentation, CNN for classification	Sensitivity, Specificity, Accuracy	Varicose ulcer	1250 images from Thoothukudi government medical college, TamilNadu, India	99.55% overall accuracy, sensitivity 95.66%, specificity 98.06%
[111]	Mask R-CNN for wound segmentation with comparison to manual experts	ANOVA, Dice Coefficients, Inter-rater ICC	CW	330 anonymized images of different wounds	Mask R-CNN showed better reproducibility (ICC = 0.99) compared to experts (ICC = 0.92), with higher processing speed

ML models were also applied to predict wound healing outcomes and risk factors (Table 19). For instance, Chairat et al. evaluated several AI models for tissue segmentation, achieving a mean IoU of 0.6964 for wound area segmentation and moderate performance for epithelialization (IoU of 0.3957) [112]. Zhao et al. focused on classifying wound depth and granulation tissue grades in diabetic wounds, achieving 84.6% accuracy in both areas [113]. Hsu et al. used an SVM-based infection assessment system to identify wound infections from 293 images, reporting an accuracy of 89.04% [114].

Some studies focused on utilizing ML to assist in clinical decision-making regarding wound management (Table 19). Nagata et al. developed classifiers such as SVM and RF to categorize wound segments from 31 images, reporting higher performance from the SVM model (Jaccard index of 68%) [115]. Similarly, Reifs et al. applied various CNN models (including ResNet50) for wound measurement, demonstrating high inter-rater reliability (0.98) and low median relative errors (2.907) in wound contour detection [116].

Finally, the integration of mobile technologies for wound care was also explored (Table 19). Zoppo et al. tested an AI-powered device called the Wound Viewer on 150 patients with various chronic wounds (DFU, PU), achieving 97% accuracy in wound bed classification [117]. This underscores the potential of AI-driven devices for remote wound monitoring and management.

**Table 19:** Summary of articles on various methods with ML techniques implemented and mobile technologies used

Article	Technology	Validation methods	Wound type	Samples	Outcomes
[112]	AI-assisted wound assessment tool with U-Net + EfficientNet-B2 and U-Net + MobilenetV2 for tissue classification and area measurement	Pixel accuracy and IoU	CW (Infection/inflammation, PU, Burn, Trauma, Diabetics)	31 images from 20 patients	Best algorithm mean IoU of 0.6964 wound area, 0.3957 epithelialization, 0.6421 granulation, and 0.1552 necrotic tissue
[113]	Bilinear CNN with VGG16 for wound depth and granulation tissue classification	Accuracy, weighted F1-score, Confusion matrix	DFU	1639 images publicly available on the Internet and from University of Massachusetts Medical School (UMMS)	84.6% accuracy for wound depth and granulation tissue amount classification
[114]	Edge-based adaptive segmentation for wound image analysis, with SVM-based infection detection module	Accuracy, True Positive Rate (TPR)	SW	293 images provided by Department of Surgery and Department of In- ternal Medicine of National Taiwan University Hospital (NTUH)	89.04% accuracy, with 76.44% TPR. Symptom detection 87.31% accuracy. Symptom assessment 83.58%
[115]	SVM and RF to classify segments into categories (wound, purpura, normal skin, etc.) using SLIC for segmentation	accuracy, weighted precision, Jaccard Index (JI), Cohen’s K	CW	31 photos from a survey in a long-term medical facility in Japan	Best performance for linear SVM (accuracy: 76%, precision: 75%, JI: 68%)
[116]	Superpixels and kmeans for wound segmentation, VGG16, InceptionResNetV2, InceptionV2, ResNet50 for classification	Intra- and inter-rater reliability, accuracy, ROC curves	CW	726 images from patients	High accuracy in Visual Computing methods. ResNet50 for classification 0.85 accuracy, area measurement Median Relative Error of 2.907
[117]	Clinical trial of AI-powered, non-invasive medical device "Wound Viewer" for wound evaluation	Kruskal-Wallis one-way analysis of variations for area and depth	Lower limb ulcers, DFU and PU	150 patients	97% accuracy compared to physicians’ WBP classifications, and tissue segmentation using devices like Visitrak and MOWA

## Discussion

The reviewed studies on wound tissue classification highlight various ML and DL approaches, with a focus on classifying different tissue types in chronic wounds, such as granulation, necrotic, and slough tissues. Mukherjee et al. combined color and texture features with an SVM model, achieving accuracies of 86.94%, 90.47%, and 75.53% for granulation, slough, and necrotic tissue, respectively [28]. Veredas et al. applied clustering algorithms and ML models, with SVM performing best, achieving 88.08% accuracy [31]. Ramachandram et al. used an EfficientNetB0-based encoder-decoder model to segment four key tissue types in a large dataset, with high scores for slough and eschar detection but struggled with epithelial tissue (F1-score of 0.253) [29]. Zahia et al. used a CNN to classify pressure injuries, achieving 92.01% accuracy, with particularly strong results for granulation and necrotic tissues [30]. Finally, Begoña et al. applied a 3D CNN, DeepMedic, for PU tissue classification, achieving excellent metrics with an AUC of 95% and DSC of 92% [32]. Together, these methods demonstrate the growing precision and effectiveness of ML/DL approaches for wound tissue classification, though challenges remain in detecting specific tissues like epithelial cells. Clinically, accurate tissue classification directly informs treatment decisions, ensuring that the right therapies are applied to specific wound types. For instance, identifying necrotic tissue accurately is essential for timely debridement, while classifying granulation tissue enables clinicians to focus on promoting tissue regeneration. This improves healing outcomes and reduces complications, such as infection or delayed wound healing.

Wound measurement techniques aim to quantify wound characteristics such as area, depth, and volume through automated image processing models, typically using methods like Mask R-CNN. These approaches ensure accuracy compared to manual assessments and improve clinical workflows. For instance, Mohammed et al. demonstrated that an AI-based tool, Swift Medical, reduced wound assessment time significantly, completing tasks in an average of 62 s [9]. Chan et al. validated the CARES4WOUNDS system for diabetic foot ulcers, showing high intra- and inter-rater reliability in wound measurement [33]. Simpson et al. evaluated digital planimetry methods for measuring systemic sclerosis ulcers, noting high reliability despite using manual segmentation, which could introduce variability [34]. In simpler approaches, Ferreira et al. employed basic computer vision techniques like grayscale conversion and Canny detection, but encountered higher error rates due to technical limitations, suggesting the need for more advanced methods [35]. In contrast, Foltynski et al. achieved high accuracy using a U-Net CNN for diabetic foot ulcers, though edge detection in chronic wounds remained challenging [36]. Niri et al. further improved wound segmentation through a DL-based method, utilizing multi-view data augmentation and 3D surface modeling to achieve significantly better DSC and IoU scores [37]. Overall, these results highlight the growing role of AI and/or DL in wound assessment, with significant improvements in time efficiency, accuracy, and measurement quality. Accurate wound measurement is essential for monitoring wound healing progress and adjusting treatment plans accordingly. Automated systems that reduce assessment time are particularly beneficial in high-volume settings, improving efficiency and ensuring consistent, real-time monitoring of wound status. This can lead to more timely interventions, reducing the risk of complications such as infection or non-healing ulcers, which are common in chronic conditions like diabetes and venous insufficiency. However, challenges such as handling irregular wound edges, metadata loss, and reliance on manual methods in some cases suggest areas for further technological development.

Wound segmentation methods focus on accurately identifying and outlining wound boundaries in images using algorithms like U-Net, Mask R-CNN, and YOLOv3. These models isolate wound regions to assist in further analysis and measurement, improving the efficiency and precision of wound care. Studies such as Anisuzzaman et al. demonstrated that YOLOv3 outperforms other models in wound localization, achieving high precision with a mAP score of 0.97 [56]. Other methods, like SVM combined with the GrabCut algorithm, achieved impressive sensitivity and specificity for pressure ulcer segmentation, while U-Net has been highlighted for its speed and efficiency in chronic wound segmentation tasks. Comparative studies of deep learning models showed that Mask R-CNN provides the highest precision for wound segmentation, while U-Net excels in recall. Hybrid techniques, such as combining LR with clustering, have also been effective in improving segmentation accuracy, while novel approaches like fuzzy spectral clustering and 3D surface modeling show significant potential for enhancing wound boundary detection. Additionally, color space selection and methods like Particle Swarm Optimization have been explored for improving segmentation accuracy, particularly in difficult cases. Real-time detection of wounds, such as diabetic foot ulcers, on mobile devices is another promising area, with models like MobileNet showing great potential for balancing speed and accuracy in clinical settings. Overall, wound segmentation enhances the precision of wound assessments, helping clinicians make more informed decisions about treatment strategies, such as whether a wound is infected or healing as expected. More accurate segmentation of wound boundaries also facilitates the calculation of wound area, depth, and volume, which are crucial parameters for assessing wound healing and determining the appropriate care, including whether advanced treatments like skin grafts or negative pressure wound therapy are needed.

Wound classification techniques utilize machine learning models such as SVM, RF, and CNNs to categorize wounds based on type, such as DFU, VU and PU. Several studies have developed advanced models to improve classification accuracy. For example, Goyal et al. used a CNN-based approach for DFU classification, achieving high sensitivity and AUC [54], while Alzubaidi et al. introduced a deep network architecture that enhanced performance in classifying DFUs [58]. Various CNN architectures, including ResNet and EfficientNet, have been applied successfully in both binary and multiclass classification tasks, achieving high accuracy and precision for identifying infection and ischemia in chronic wounds. Hybrid models that combine deep features with hand-crafted features have also shown promising results. Traditional machine learning models like RF and DT have been effective in identifying high-risk patients and classifying wounds from clinical data. Additionally, novel approaches like transfer learning, explainable AI, and YOLO-based models have demonstrated high performance in wound detection and classification. These classification methods supports early diagnosis, optimal treatment planning, and identification of complications. For instance, correctly identifying diabetic foot ulcers or pressure ulcers helps guide targeted treatments such as offloading pressure or managing infection risks. The use of AI-based classification can assist clinicians in identifying high-risk patients earlier, allowing for proactive care interventions, thus reducing the risk of more severe complications like amputations or chronic non-healing wounds.

Recent advancements in ML and DL have significantly enhanced wound assessment and healing prediction, with promising implications for clinical decision-making. For instance, the Reduced ResNet-18 model achieved an IoU rate above 0.5 in detecting granulation tissue in diabetic foot ulcers, showcasing the potential of lightweight networks in resource-constrained settings. Similarly, Liu et al. employed a DenseNet framework to assess wound attributes, attaining accuracy and F1 scores over 0.8, highlighting the effectiveness of sophisticated neural architectures [93]. Clinically, models that predict wound healing potential can help clinicians make informed decisions about treatment duration and intensity, particularly for chronic wounds. For example, accurate detection of granulation tissue indicates the healing phase, which can guide decisions about whether to continue the current treatment regimen or explore more advanced therapies.

Incorporating patient empowerment, Chen et al. demonstrated that smartphone-based systems could accurately evaluate surgical wounds with over 90% accuracy, facilitating self-management in chronic wound care [95]. Additionally, studies utilizing NB classifiers showed improved predictions for surgical site infections (SSIs) and hard-to-heal diabetic foot ulcers, with an impressive AUC of 0.864 achieved by Wang et al. [98]. From a clinical perspective, smartphone-based systems enable patients to monitor their wounds in real-time, leading to improved self-management and earlier detection of complications. This can be especially beneficial for patients with chronic conditions like diabetes, where ongoing wound care is necessary to prevent complications such as infections or amputations. Empowering patients to track their healing progress fosters adherence to treatment plans and enhances overall outcomes.

Hybrid approaches that combine structured and unstructured data further enhance predictive accuracy, as shown by Song et al. (2021), where integrating clinical notes significantly improved outcomes for wound infection-related hospitalizations [101]. Clinically, these hybrid models offer a comprehensive understanding of the patient’s overall health status, allowing for more personalized care and better predictions of wound healing and complications. By integrating clinical data, such as comorbidities, treatment history, and wound characteristics, these models provide clinicians with more accurate risk assessments, guiding timely interventions and improving patient outcomes. Collectively, these studies underscore the transformative potential of ML and DL in wound care management, paving the way for more personalized treatment strategies. Continued research is needed to refine these models for broader clinical adoption, ultimately improving patient outcomes.

### Relationship Between AI Models and Traditional Wound Assessment Tools

Advances in ML and DL have transformed chronic wound care, serving as both an alternative and a complement to traditional tools such as the PUSH, BWAT assessment scales, and digital planimetry. While these traditional methodologies, grounded in manual or semi-automated assessments, have been validated, they exhibit inherent limitations in terms of accuracy, efficiency, and subjectivity-limitations that AI seeks to address (Table 20).

**Table 20:** Comparison of different aspects between AI models and traditional wound assessment tools

Aspect	Traditional Tools	AI Models
Precision	Dependent on the evaluator	Consistent and automated
Subjectivity	High, variable between users	Fast, Low, based on objective and reproducible data
Speed	Slow manual evaluations	Fast, real-time processing
Predictions and Outcomes	Limited	Advanced, includes complications and progression
Generalization	Specific to certain wound types	Broad clinical applicability

Traditional tools rely heavily on clinical judgment, introducing variability into wound evaluations. Measurement devices like ruler or digital planimetry (e.g., Visitrak) improve precision by providing semi-automated wound area measurements. However, such tools still depend on user input for tracing wound boundaries, making them susceptible to inter- and intra-rater variability. AI-based tools like U-Net and AutoTrace, by contrast, enhance precision through full automation, segmenting wound areas and classifying tissue types consistently, even in chronic or irregular wound scenarios.

Furthermore, AI-based tools process data in real time. For example, Swift Medical has demonstrated a significant reduction in evaluation time, completing assessments in an average of 62 s per wound. This capability not only streamlines clinical workflows but also enables higher patient throughput without compromising accuracy.

Whereas traditional tools predominantly focus on static assessments, AI models integrate longitudinal data to predict clinical risks such as delayed healing or future infections. This predictive capability offers valuable insights that inform clinical decision-making and enable tailored treatment strategies. Additionally, while tools like PUSH, BWAT, and Visitrak are designed for specific wound types or require manual interventions, AI models exhibit broader applicability, encompassing diabetic ulcers, vascular ulcers, and other chronic wound etiologies.

Despite these advancements, the integration of AI models into clinical practice requires addressing certain challenges. Comprehensive clinical validation is essential, requiring studies that evaluate AI performance in real-world contexts. Moreover, ensuring healthcare professionals are adequately trained to use these tools effectively is critical. Finally, accessible technological infrastructure must be developed to facilitate the adoption of AI systems, particularly in resource-limited settings.

### Studies Limitations

The evidence included in the review faces several limitations. One significant issue is the limited generalizability of many ML and DL models due to small or highly specific datasets. Most studies rely on curated wound datasets that may not reflect the diversity of real-world conditions, limiting their broader clinical applicability. Furthermore, many models are tested under controlled environments or specific conditions making it difficult to generalize their effectiveness across other wound types or patient populations.

Another limitation is the variability in performance metrics used across studies, such as accuracy, AUC, F1-score, and DSC, which complicates direct comparisons between different approaches. Moreover, some models face difficulties in accurately identifying certain types of tissues, suggesting that the current algorithms may not be equally effective at recognizing all features within a wound. This limitation can lead to incomplete or incorrect evaluations, particularly when dealing with more chronic wound types. To improve detection, models need to be specifically trained to recognize designated tissue types. If certain tissues are not included in the training set, the model will be unable to identify them during its analysis, leading to potential gaps in assessment.

The reliance on manual segmentation or annotations for model training and evaluation also introduces variability, especially in wound measurement and segmentation tasks, where human error can affect the ground truth. Additionally, many studies focus on retrospective data analysis rather than prospective trials, which may limit the assessment of model performance in real-time clinical settings.

Of the 93 studies included in this review, only 21 explicitly addressed wounds with irregular edges or multiple/noncontiguous lesions. This gap highlights a significant deficiency in the existing literature, especially considering that such characteristics are common in clinical practice, particularly among patients with venous ulcers or pressure injuries. These types of wounds present unique challenges for diagnosis and treatment, which limits the applicability of the AI tools reviewed to broader clinical scenarios. The absence of models and tools specifically designed to address these conditions underscores the need for future research focusing on such wounds to improve their management and the effectiveness of AI-based methods.

Lastly, while some models integrate XAI tools, the lack of interpretability in most ML/DL algorithms remains a concern, particularly in clinical environments where understanding the decision-making process is critical. This limits the trust and adoption of these technologies by healthcare professionals. More research is needed to refine these models, improve data diversity, and establish consistent evaluation standards to better validate their clinical utility.

### Future Research

Future research should focus on addressing the limitations of current AI and/or ML models, particularly their performance on diverse and chronic wound types, such as wounds with irregular edges or mixed tissue types. Developing larger and more diverse datasets, ensuring better data annotation quality, and creating models that generalize well to various clinical settings will be important next steps. A crucial aspect of this is the inclusion of real-world data in model training and validation. AI models trained on curated, controlled datasets may not perform adequately in the diverse, heterogeneous conditions encountered in everyday clinical practice. Incorporating wounds with varied geometries, sizes, and etiologies into training datasets is essential for developing robust, adaptable models that can handle the complexity of real-world scenarios.

Moreover, research should explore hybrid models that combine ML with traditional clinical data, including patient demographics, medical history, and environmental factors. Such approaches could enhance model performance by incorporating a broader context, improving decision-making in multifactorial wound care. Additionally, methods to improve the interpretability of AI algorithms will be vital for healthcare professionals, ensuring that AI tools are not seen as "black boxes" but as supportive, transparent systems that can explain their decision-making process.

There is also a need for prospective, real-world studies to validate the clinical efficacy of these AI tools and determine their long-term impact on patient outcomes. These studies should involve a variety of wound types and clinical contexts, reflecting the true diversity of cases seen in clinical practice. Furthermore, research into the cost-effectiveness of AI-driven wound care, as well as strategies for integrating these tools into existing healthcare infrastructures, will be critical for ensuring the sustainable implementation and widespread adoption of AI in wound management. This research will not only validate the clinical utility of AI tools but also ensure their accessibility and effectiveness across different healthcare settings.

## Conclusion

The studies reviewed illustrate the transformative potential of ML and DL approaches in wound assessment, classification, and measurement. Techniques such as SVM, CNN, and advanced hybrid models have achieved impressive accuracy and efficiency in distinguishing between different tissue types, quantifying wound characteristics, and predicting healing outcomes. For example, models like the EfficientNetB0 and DeepMedic have shown high performance in segmenting and classifying wound tissues, while smartphone applications enable patients to participate actively in their care. However, challenges remain, particularly in accurately identifying specific tissue types like epithelial cells and managing chronic wound boundaries. Continued advancements in algorithm development, as well as the integration of structured and unstructured data, are essential for further enhancing the precision of wound care technologies. Ultimately, these innovations promise to improve clinical workflows, support personalized treatment strategies, and lead to better patient outcomes in wound management.

**Supplementary information** The data that supports the findings of this study are available in the supplementary material of this article.

## Supplementary Information

Below is the link to the electronic supplementary material.

Supplementary file 1 (docx 117 KB)

## Data Availability

The data used and analysed during the current study are available from the corresponding author on reasonable request.

## Data and materials availability

No datasets were generated or analysed during the current study.
