# Supplementary material for: Artificial Intelligence Methods for Diagnostic and Decision-Making Assistance in Chronic Wounds: A Systematic Review
Source: J Med Syst. 2025 Feb 19;49(1):29. doi: 10.1007/s10916-025-02153-8 (PMC11839728; doi:10.1007/s10916-025-02153-8)
Supplement: Supplementary file 1 — (docx 117 KB) [file 10916_2025_2153_MOESM1_ESM.docx]

SUPPLEMENTARY MATERIAL

This supplementary material provides two essential components related to the systematic review on chronic wound diagnosis. First, it includes the PRISMA checklist, which outlines the criteria used for reporting systematic reviews and meta-analyses. Second, it presents the quality assessment based on the QUADAS-2 framework, evaluating the risk of bias across included studies. These tools are designed to ensure transparency, rigor, and consistency in the review process, contributing to the reliability of the findings and their implications for future research and clinical applications in chronic wound care.

**PRISMA CHECKLIST**

| **TITLE** | | |
| --- | --- | --- |
| Title | 1 | Technological methods to assess the decision diagnosis in chronic wounds: a systematic review |
| **ABSTRACT** | | |
| Abstract | 2 | Chronic wounds, defined as wounds that takes more than 6 weeks to heal (ref-1), are a widespread and debilitating health issue affecting millions of people worldwide. These non-healing wounds often result from underlying conditions such as diabetes, venous insufficiency, arterial diseases, pressure ulcers, and more, and can cause significant pain, reduce quality of life, and impose a heavy economic burden on both individuals and healthcare systems. Accurate and timely diagnosis is crucial for effective wound management and preventing severe complications. In recent years, advancements in technology have revolutionized chronic wounds diagnosis, empowering healthcare professionals with innovative tools to enhance precision, speed, and patient outcomes. In this systematic review, we explore the transformative role of technology in wound care and its potential to improve the lives of those affected by chronic wounds. Many articles from different databases (ACM, IEEE, Pubmed, Scopus, Web of Science) published between 2013 and 2023 and related to diagnosis through complex computational methods in images of chronic wounds have been included. Articles on other types of samples different from images of chronic wounds, review articles and non-English or non-Spanish texts were excluded. Outcomes include clinical use, wound measurement statistics, hospital system integration, and other advantages and challenges. The search identified 2791 articles. 93 full-text studies were eligible for the final analysis. |
| **INTRODUCTION** | | |
| Rationale | 3 | Chronic wounds are wounds that do not heal within the expected timeframe of the body’s natural healing process, typically defined as wounds that fail to heal within 6 weeks. These wounds can result from various underlying factors, such as the age of the patient and the presence of underlying chronic comorbidities. The prevalence of chronic wounds is more significant than most people realize, making it a silent epidemic. According to the World Health Organization (WHO), an estimated 4.5 million people worldwide suffer from chronic wounds at any given time. This number is expected to rise due to the aging population, the increasing prevalence of conditions like diabetes and obesity, and a growing number of people with reduced mobility. Diabetes, in particular, is a leading cause of chronic wounds. Diabetic foot ulcers alone affect approximately 15% of individuals with diabetes during their lifetime, and it’s estimated that 1 in 4 diabetics will experience a foot ulcer at some point. The impact of chronic wounds on patient’s lives cannot be overstated. Besides the physical pain and discomfort, chronic wounds often lead to emotional distress and decreased mental well-being. Patients may experience anxiety, depression, social isolation, and a loss of independence due to restricted mobility. The risk of infection is another significant concern with chronic wounds, as they create an entry point for bacteria and can lead to amputations, further exacerbating the physician and psychological burden on affected individuals.  Chronic wounds place a substantial financial burden on healthcare systems and society. Treatment costs for managing chronic wounds are high, often requiring specialized wound care products, prolonged hospital stays, and frequent follow-up visits. It is often believed that the use of wound dressings per se is the major cost driver in wound management, whereas in fact, nursing time and hospital costs are together responsible for around 80-85% of the total cost. In Europe only, it is estimated that the cost of treating diabetic foot ulcers alone exceeds 4€ billion annually. Additionally, chronic wounds can lead to decreased productivity and lost workdays for patients and caregivers. Wound management is estimated to account for over 50% of community nurse time in European studies, with patients often having three or more home health visits per week. The cost of reduced quality of life, combined with the economic impact, underscores the urgency of finding effective prevention and treatment strategies.  Early detection and proper management are crucial in addressing chronic wounds effectively. A multidisciplinary approach that involves wound care specialists, physicians, nurses, dietitians, and other healthcare professionals is often necessary. Preventive measures are equally important, especially for high-risk individuals. For diabetics, strict glycerin control, foot care, and regular medical check-ups can significantly reduce the risk of developing chronic wounds. Proper nutrition, maintaining a healthy weight, and regular exercise can help prevent wounds caused by arterial and venous insufficiency.  The fight against chronic wounds is ongoing, and innovative approaches are emerging to improve patient outcomes. Researchers are exploring advanced technologies to transform various aspects of healthcare, including chronic wound diagnosis and management and provide clinicians with user-friendly tools to capture wound images, measure dimensions, and track healing progress over time. This data helps in tailoring personalized treatment plans and identifying potential complications early on. With Artificial Intelligence (AI) and Machine Learning (ML) algorithms is possible to analyze vast amounts of wound-related data, identify patterns, and assist clinicians in making informed decisions, including the early detection of infection reducing the risk of complications. Telemedicine and digital health solutions are also being utilized to monitor and manage chronic wounds remotely, making healthcare more accessible to patients, especially those in rural or underserved areas.  Technology has become a game-changer in the field of chronic wound diagnosis. From advanced imaging modalities to mobile applications, AI-driven algorithms among other innovations are empowering healthcare professionals with the tools needed to make accurate and timely diagnosis, tailor individualized treatments plans, and improve patient outcomes. As technology continues to evolve, we can expect even more ground-breaking developments in wound care, further enhancing the quality of life for those living with chronic wounds. However, it is essential to strike a balance between technology and human touch, ensuring that compassionated and skilled wound care remains at the core of every patient’s journey toward healing and recovery. |
| Objectives | 4 | The main objective of this systematic review is complete information on new and emerging techniques for the assessment of chronic wounds, as well as the monitoring and follow-up of the healing process in the existing literature. |
| **METHODS** | | |
| Eligibility criteria | 5 | Inclusion criteria where diagnosis, follow-up, monitoring, healing, assessment, treatment, identification, management, evaluation, recognition, classification or segmentation of wound, ulcer, skin lesion or skin damage using AI, ML, Deep Learning (DL), Convolutional Neural Networks (CNN), Deep Neural Network, Supervised Learning, Transfer Learning, delineation, Support Vector Machines (SVM), clustering, automatic detection or computer assisted. Exclusion criteria were other types of non-superficial wounds and non RGB images such as radiographs, ultrasound, computed tomography, and magnetic resonance imaging. Skin lesions such as melanomas or skin cancer with interesting methods have been screened to leave as "searchable". Also, review articles, letters to editors or editorials, non-English and non-Spanish articles or articles without full texts are excluded. |
| Information sources | 6 | We conducted electronic searches for eligible studies within each of the following databases:  Association for Computing Engineering (ACM) (Jan 2013 to May 2023)  Institute of Electrical and Electronics Engineers (IEEE) (1^st^ Jan 2013 to 22^nd^ May 2023)  Pubmed (1^st^ Jan 2013 to 22^nd^ May 2023)  Scopus (1^st^ Jan 2013 to 22^nd^ May 2023)  Web of science (1^st^ Jan 2013 to 22^nd^ May 2023) |
| Search strategy | 7 | For all databases we used the advanced search interface with the following words and statements appearing in the title or abstract:  wound OR  ulcer OR  skin lesion OR  skin damage OR  AND  diagnosis OR  follow up OR  monitoring OR  healing OR  assessment OR  treatment OR  identification OR  management OR  evaluation OR  recognition OR  classification OR  segmentation OR  AND  artificial intelligence OR  machine learning OR  deep learning OR  convolutional neural network OR  deep neural network OR  supervised learning OR  transfer learning OR  delineation OR  support vector machine OR  clustering OR  automatic detection OR  computer assisted OR  For ACM we used the search syntax:  “Abstract:(wound OR ulcer OR "skin lesion" OR "skin damage") AND Abstract:(diagnosis OR "follow up" OR monitoring OR healing OR assessment OR treatment OR identification OR diagnosing OR management OR evaluation OR recognition OR classification OR segmentation) AND Abstract:("artificial intelligence" OR "machine learning" OR "deep learning" OR "convolutional neural network" OR "deep neural network" OR "supervised learning" OR "transfer learning" OR "delineation" OR "support vector machine" OR clustering OR automatic detection OR "computer assisted")”  for the abstract and the following syntax for the title:  “Title:(wound OR urlcer OR "skin lesions" OR "skin damage") AND Title:(diagnosis OR "follow up" OR monitoring OR healing OR assessment OR treatment OR identification OR management OR evaluation OR recognition OR classification OR segmentation) AND Title:("artificial intelligence" OR "machine learning" OR "deep learning" OR "convolutional neural network" OR "deep neural network" OR "supervised learning" OR "transfer learning" OR "delineation" OR "support vector machine" OR clustering OR "automatic detection" OR "computer assisted")”  The filters for the date have been made through the search wizard and a restriction of publication date between January 2013 and May 2023 has been applied. The search results were 3 results in the title search and 30 results in the abstract search, giving a total of 33 results.  For IEEE we used the search syntax:  ("Abstract":"wound" OR "Abstract":"ulcer" OR "Abstract":"skin lesion" OR "Abstract":"skin damage") AND ("Abstract":"diagnosis" OR "Abstract":"follow up" OR "Abstract":"monitoring" OR "Abstract":"healing" OR "Abstract":"assessment" OR "Abstract":"treatment" OR "Abstract":"identification" OR "Abstract":"management" OR "Abstract":"evaluation" OR "Abstract":"recognition" OR "Abstract":"classification" OR "Abstract":"segmentation") AND ("Abstract":"artificial intelligence" OR "Abstract":"machine learning" OR "Abstract":"deep learning" OR "Abstract":"convolutional neural network" OR "Abstract":"deep neural network" OR "Abstract":"supervised learning" OR "Abstract":"transfer learning" OR "Abstract":"delineation" OR "Abstract":"support vector machines" OR "Abstract":"clustering" OR "Abstract":"automatic detection" OR "Abstract":"computer assisted")  for the abstract and the following syntax for the title:  “("Document Title":"wound" OR "Document Title":"ulcer" OR "Document Title":"skin lesion" OR "Document Title":"skin damage") AND ("Document Title":"diagnosis" OR "Document Title":"follow up" OR "Document Title":"monitoring" OR "Document Title":"healing" OR "Document Title":"assessment" OR "Document Title":"treatment" OR "Document Title":"identification" OR "Document Title":"management" OR "Document Title":"evaluation" OR "Document Title":"recognition" OR "Document Title":"classification" OR "Document Title":"segmentation") AND ("Document Title":"artificial intelligence" OR "Document Title":"machine learning" OR "Document Title":"deep learning" OR "Document Title":"convolutional neural network" OR "Document Title":"deep neural network" OR "Document Title":"supervised learning" OR "Document Title":"transfer learning" OR "Document Title":"delineation" OR "Document Title":"support vector machines" OR "Document Title":"clustering" OR "Document Title":"automatic detection" OR "Document Title":"computer assisted")”  The filters for the date have been made through the search wizard and a restriction of publication date between 2013 and 2023 has been applied. The search results were 36 results in the title search and 58 results in the abstract search, giving a total of 94 results.  For Pubmed we used the search syntax:  “((wound[Title/Abstract]) OR (ulcer[Title/Abstract]) OR (skin lesion[Title/Abstract]) OR (skin damage[Title/Abstract])) AND ((diagnosis[Title/Abstract]) OR (follow up[Title/Abstract]) OR (monitoring[Title/Abstract]) OR (healing[Title/Abstract]) OR (assessment[Title/Abstract]) OR (treatment[Title/Abstract]) OR (identification[Title/Abstract]) OR (management[Title/Abstract]) OR (evaluation[Title/Abstract]) OR (recognition[Title/Abstract]) OR (classification[Title/Abstract]) OR (segmentation[Title/Abstract])) AND ((artificial intelligence[Title/Abstract]) OR (machine learning[Title/Abstract]) OR (deep learning[Title/Abstract]) OR (convolutional neural network[Title/Abstract]) OR (deep neural network[Title/Abstract]) OR (supervised learning[Title/Abstract]) OR (transfer learning[Title/Abstract]) OR (delineation[Title/Abstract]) OR (support vector machine[Title/Abstract]) OR (clustering[Title/Abstract]) OR (automatic detection[Title/Abstract]) OR (computer assisted[Title/Abstract]))”  The filter for the date and type of document has been made through the search wizard and were the following: Books and Documents, Case Reports, Classical Article, Clinical Study, Clinical Trial, Comparative Study, Corrected and Republished Article, Meta-Analysis, Newspaper Article, Portrait, Randomized Controlled Trial, Technical Report, in the last 10 years. The total number of results were 529.  For Scopus we used the search syntax:  TITLE-ABS ( ( wound OR ulcer OR {skin lesion} OR {skin damage} ) AND ( diagnosis OR {follow up} OR monitoring OR healing OR assessment OR treatment OR identification OR management OR evaluation OR recognition OR classification OR segmentation ) AND ( {artificial intelligence} OR {machine learning} OR {deep learning} OR {convolutional neural network} OR {deep neural network} OR {supervised learning} OR {transfer learning} OR delineation OR {support vector machine} OR clustering OR {automatic detection} OR {computer assisted} ) )  The filter for the date and type of document has been made through the search wizard and were the following: Article, Conference paper, Book chapter, Note, Erratum, Retracted, Undefined, Letter, Short Survey, Editorial, Data paper, Book limited between 2013 and 2023. The advanced syntax is the following:  “PUBYEAR > 2013 AND PUBYEAR < 2024 AND ( LIMIT-TO ( DOCTYPE , "ar" ) OR LIMIT-TO ( DOCTYPE , "cp" ) OR LIMIT-TO ( DOCTYPE , "ch" ) OR LIMIT-TO ( DOCTYPE , "er" ) OR LIMIT-TO ( DOCTYPE , "no" ) OR LIMIT-TO ( DOCTYPE , "tb" ) OR LIMIT-TO ( DOCTYPE , "sh" ) OR LIMIT-TO ( DOCTYPE , "le" ) OR LIMIT-TO ( DOCTYPE , "dp" ) OR LIMIT-TO ( DOCTYPE , "Undefined" ) OR LIMIT-TO ( DOCTYPE , "ed" ) )”  The total number of results were 1.258.  For Web of Science we used the search syntax:  “(TI=(wound) OR TI=(ulcer) OR TI=("skin lesion") OR TI=("skin damage")) AND (TI=(diagnosis) OR TI=("follow up") OR TI=(monitoring) OR TI=(healing) OR TI=(assessment) OR TI=(treatment) OR TI=(identification) OR TI=(management) OR TI=(evaluation) OR TI=(recognition) OR TI=(classification) OR TI=(segmentation)) AND (TI=("artificial intelligence") OR TI=("machine learning") OR TI=("deep learning") OR TI=("convolutional neural networks") OR TI=("deep neural network") OR TI=("supervised learning") OR TI=("transfer learning") OR TI=(delineation) OR TI=("support vector machine") OR TI=(clustering) OR TI=("automatic detection") OR TI=("computer assisted")) OR (AB=(wound) OR AB=(ulcer) OR AB=("skin lesion") OR AB=("skin damage")) AND (AB=(diagnosis) OR AB=("follow up") OR AB=(monitoring) OR AB=(healing) OR AB=(assessment) OR AB=(treatment) OR AB=(identification) OR AB=(management) OR AB=(evaluation) OR AB=(recognition) OR AB=(classification) OR AB=(segmentation)) AND (AB=("artificial intelligence") OR AB=("machine learning") OR AB=("deep learning") OR AB=("convolutional neural networks") OR AB=("deep neural network") OR AB=("supervised learning") OR AB=("transfer learning") OR AB=(delineation) OR AB=("support vector machine") OR AB=(clustering) OR AB=("automatic detection") OR AB=("computer assisted"))”  The filter for the date and type of document has been made through the search wizard and were all types except Review limited between years 2013 and 2023. The total number of results were 877 articles. |
| Selection process | 8 | Citations identified from the literature searches were imported to Excel and duplicates were removed. Three reviewers independently screened the citations with explanatory comments on the decision when necessary: first one screened all citations and the rest reviewed half each. The inclusion criteria were refined after reviewing the first 100 citations. Disagreements about eligibility were resolved through discussion. |
| Data collection process | 9 | Data points such as titles, authors, abstracts, year of publication, and article types were systematically extracted. Three independent reviewers assessed the included studies. One reviewer analyzed all studies, the other two reviewers split the remaining studies equally. A clear justification for exclusions was provided. In cases of disagreement, reviewers discussed until consensus was reached. No specific automation tools were used. |
| Data items | 10a | The focus was on clinical utility, including ML models employed and outcomes such as: Tissue classification, Wound measurement, Image segmentation, Wound classification, Healing prediction. The outcomes included metrics like accuracy, F1-score, sensitivity, specificity, and Dice Similarity Coefficient (DSC), depending on the tasks. |
|  | 10b | Variables like patient data, wound image samples, and clinical test results were considered.  Missing or unclear data was addressed through validation by three independent reviewers. |
| Study risk of bias assessment | 11 | The QUADAS-2 framework was adapted for assessing risk of bias. The framework assessed domains like: Patient Selection, Index Test (e.g., algorithms, training, and validation), Reference Standard (e.g., ground truth data), Flow and Timing, Number of Reviewers: Three independent reviewers participated in the assessment. We didn’t use automation tools. |
| Effect measures | 12 | Effect measures included: Accuracy, Sensitivity/Recall, Specificity, Precision, F1-score, Dice Similarity Coefficient (DSC), Intersection over Union (IoU), Matthews Correlation Coefficient (MCC), AUC-ROC for model performance evaluation. |
| Synthesis methods | 13a | Studies were synthesized based on task types: tissue classification, measurement, segmentation, wound classification, and healing prediction. |
|  | 13b | Conversion to RGB image formats. Handling missing annotations or validation |
|  | 13c | Results were tabulated and displayed using structured tables summarizing models, metrics, wound types, and outcomes. |
|  | 13d | Comparative analysis of deep learning models (e.g., U-Net, Mask R-CNN, YOLOv3)  Novel methods (e.g., fuzzy clustering, DBSCAN, morphological operations)  Models were chosen based on metrics like AUC, DSC, and IoU. |
|  | 13e | Heterogeneity was explored through comparative studies of different model architectures and metrics. |
|  | 13f | Sensitivity analyses were not applied. |
| Reporting bias assessment | 14 | Reporting bias was primarily evaluated through the Reference Standard domain of the QUADAS-2 framework. |
| Certainty assessment | 15 | Issues included inadequate description of annotation methods and absence of inter-rater reliability metrics. |

*From:* Page MJ, McKenzie JE, Bossuyt PM, Boutron I, Hoffmann TC, Mulrow CD, et al. The PRISMA 2020 statement: an updated guideline for reporting systematic reviews. BMJ 2021;372:n71. doi: 10.1136/bmj.n71

QUALITY ASSESSMENT – QUADAS 2

# Analysis of Chronic Wound Images Using Factorization-Based Segmentation and Machine Learning Methods

**Key Findings:**

- Developed a pipeline combining preprocessing (color correction, noise removal), factorization-based segmentation, and machine learning classifiers (SVM, RF, MLP).
- Achieved high accuracy for distinguishing pressure ulcers and leg ulcers in a dataset of 59 images, but performance was limited by dataset size and diversity.

**Recommendations:**

- Expand the dataset to include more varied wound types and larger sample sizes.
- Evaluate in real-world clinical settings to assess robustness.

| **Domain** | **Risk of Bias** | **Applicability** |
| --- | --- | --- |
| Patient Selection | Low | Low |
| Index Test | Low | Low |
| Reference Standard | High | Uncertain |
| Flow and Timing | Low | Low |

# Varicose Ulcer (C6) Wound Image Tissue Classification Using Multidimensional CNNs

**Key Findings:**

- Employed multidimensional CNNs for tissue classification (granulation, slough, necrotic, epithelial) of varicose ulcer images with preprocessing and segmentation steps.
- Achieved an impressive accuracy of 99.55% but relied heavily on a single dataset sourced from a limited clinical population.

**Recommendations:**

- Incorporate external validation datasets to test generalizability.
- Consider reducing reliance on preprocessing to better reflect real-world variability.

| **Domain** | **Risk of Bias** | **Applicability** |
| --- | --- | --- |
| Patient Selection | Low | Low |
| Index Test | Low | Low |
| Reference Standard | High | Uncertain |
| Flow and Timing | Low | Low |

# Wound Image Evaluation with Machine Learning

**Key Findings:**

- Utilized k-means clustering for segmentation and SVM, NN, and RF classifiers for tissue classification in pressure ulcer images.
- Achieved high accuracy rates (SVM: 88.08%, RF: 87.37%) and demonstrated robust statistical validation but lacked prospective real-world testing.

**Recommendations:**

- Validate algorithms on larger datasets with varied demographics.
- Explore real-time integration for clinical decision support.

| **Domain** | **Risk of Bias** | **Applicability** |
| --- | --- | --- |
| Patient Selection | Low | Low |
| Index Test | Low | Low |
| Reference Standard | Low | Low |
| Flow and Timing | Low | Low |

# Fully Automatic Wound Segmentation with Deep Convolutional Neural Networks

**1. Patient Selection**

- **Assessment**: Patients were from a clinical wound center, and the dataset comprised 1,109 foot ulcer images from 889 patients. The inclusion criteria ensured clinical relevance, but the lack of detail on randomization or consecutive sampling may introduce selection bias.
- **Risk of Bias**: Moderate
- **Applicability**: High, as the dataset is representative of foot ulcer cases.

**2. Index Test**

- **Assessment**: MobileNetV2 was applied with clear technical details, but no mention of blinding between the segmentation process and the ground truth labels.
- **Risk of Bias**: Moderate
- **Applicability**: High, as MobileNetV2 is well-suited for wound segmentation tasks.

**3. Reference Standard**

- **Assessment**: Segmentation masks were created and reviewed by wound care specialists. No information is provided about their blinding.
- **Risk of Bias**: Moderate
- **Applicability**: High, given the clinical relevance of manual annotations.

**4. Flow and Timing**

- **Assessment**: All images appear included in analysis, but the timing between data collection and annotation is not clarified.
- **Risk of Bias**: Low
- **Applicability**: High, as the workflow aligns with clinical applications.

**Summary Table**

| **Domain** | **Risk of Bias** | **Applicability** |
| --- | --- | --- |
| Patient Selection | Moderate | High |
| Index Test | Moderate | High |
| Reference Standard | Moderate | High |
| Flow and Timing | Low | High |

**Recommendations**:

1. Ensure randomization or consecutive sampling in future datasets.
2. Clearly report blinding of annotators to reduce bias.
3. Include detailed timing protocols for improved reproducibility.

# Pressure Ulcer Diagnosis with Superpixel Segmentation

**1. Patient Selection**

- **Assessment**: The dataset includes 2,836 images for tissue classification and 2,893 for segmentation. Patient demographics or inclusion/exclusion criteria are unclear, limiting the generalizability of findings.
- **Risk of Bias**: Moderate
- **Applicability**: Moderate, as the dataset targets pressure ulcers but may lack diversity.

**2. Index Test**

- **Assessment**: Five deep learning models were used, with detailed descriptions. However, the labeling process by surgeons was not blinded to model outputs.
- **Risk of Bias**: Moderate
- **Applicability**: High, given the focus on pressure ulcer segmentation.

**3. Reference Standard**

- **Assessment**: Images were co-labeled by surgeons, but lack of blinding raises concerns about bias.
- **Risk of Bias**: High
- **Applicability**: High, given surgeon expertise.

**4. Flow and Timing**

- **Assessment**: Dataset composition and exclusions are detailed, but the timing of image acquisition relative to annotations is unclear.
- **Risk of Bias**: Low
- **Applicability**: Moderate, due to potential selection effects.

**Summary Table**

| **Domain** | **Risk of Bias** | **Applicability** |
| --- | --- | --- |
| Patient Selection | Moderate | Moderate |
| Index Test | Moderate | High |
| Reference Standard | High | High |
| Flow and Timing | Low | Moderate |

**Recommendations**:

1. Provide detailed patient demographics to ensure dataset diversity.
2. Establish blinding procedures during annotation.
3. Clarify timing protocols to enhance study transparency.

# Internet-Based Wound Measurement with CNNs

**1. Patient Selection**

- **Assessment**: The dataset includes 565 images from multiple sources but lacks information on patient demographics or inclusion criteria.
- **Risk of Bias**: High
- **Applicability**: Moderate, given its relevance to diabetic foot ulcers but limited diversity.

**2. Index Test**

- **Assessment**: A U-Net CNN model was used with clear technical descriptions, but potential biases exist without blinding during segmentation validation.
- **Risk of Bias**: Moderate
- **Applicability**: High, due to its suitability for wound area measurement.

**3. Reference Standard**

- **Assessment**: Ground truth masks were generated, but the lack of blinding and specifics about the annotation process raises concerns.
- **Risk of Bias**: High
- **Applicability**: High, as annotations are clinically relevant.

**4. Flow and Timing**

- **Assessment**: Detailed steps for preprocessing and segmentation are provided, but there is limited information on exclusions or time intervals.
- **Risk of Bias**: Low
- **Applicability**: High, as the study aligns well with clinical practices.

**Summary Table**

| **Domain** | **Risk of Bias** | **Applicability** |
| --- | --- | --- |
| Patient Selection | High | Moderate |
| Index Test | Moderate | High |
| Reference Standard | High | High |
| Flow and Timing | Low | High |

**Recommendations**:

1. Broaden the dataset to include a more representative patient population.
2. Implement blinding procedures during annotation.
3. Document patient and annotation timing details.

# Wound Image Evaluation Using ML - Veredas et al. (2015)

#### **1. Patient Selection**

- A small dataset (113 photographs) from home-care patients, focusing on sacrum and hip pressure ulcers.
- Patients selected by clinicians based on tissue variety, but lack of broader representativeness.
- **Risk of Bias:** Moderate
- **Applicability:** Moderate

#### **2. Index Test and Reference Standard**

- Employed k-means clustering and three ML classifiers (SVM, NN, RF).
- Ground truth provided by clinicians, but the process for labeling is not blinded or validated across raters.
- **Risk of Bias:** Moderate
- **Applicability:** High

#### **3. Flow and Timing**

- No mention of follow-up imaging or temporal assessment.
- Exclusions were justified based on spurious image quality issues.
- **Risk of Bias:** Low
- **Applicability:** Moderate

#### **4. Overall Bias and Applicability**

- **Bias:** Moderate
- **Applicability:** Moderate

| **Domain** | **Risk of Bias** | | | | **Applicability** | | | | | |  |  |
| --- | --- | --- | --- | --- | --- | --- | --- | --- | --- | --- | --- | --- |
| **Patient Selection** | | | Moderate | | | | | Moderate | | | |  |
| **Index Test/Ref Standard** | | | | | | | Moderate | | | High | | |
| **Flow and Timing** | | | | Low | | Moderate | | |  |  |  |  |
| **Overall Bias** | | Moderate | | | | Moderate | | |  |  |  |  |

# Image Segmentation with Transfer Learning and Fast R-CNN - Huang et al. (2022)

#### **1. Patient Selection**

- Dataset includes diabetic foot ulcers with object detection using Fast R-CNN.
- Pre-labeled images provided by clinicians without specifying validation steps.
- **Risk of Bias:** Moderate
- **Applicability:** High

#### **2. Index Test and Reference Standard**

- Fast R-CNN with transfer learning, trained on annotated wound images.
- Physician-labeled data served as the reference but lacks inter-rater agreement assessment.
- **Risk of Bias:** Moderate
- **Applicability:** High

#### **3. Flow and Timing**

- Real-time assessment capability but lacks temporal detail regarding post-intervention follow-ups.
- Exclusions not mentioned explicitly.
- **Risk of Bias:** Moderate
- **Applicability:** Moderate

#### **4. Overall Bias and Applicability**

- **Bias:** Moderate
- **Applicability:** High

| **Domain** | **Risk of Bias** | | | | **Applicability** | | | | | | | |  |  |
| --- | --- | --- | --- | --- | --- | --- | --- | --- | --- | --- | --- | --- | --- | --- |
| **Patient Selection** | | | Moderate | | | | | High | | |  |  |  |  |
| **Index Test/Ref Standard** | | | | | | | Moderate | | | | | High | | |
| **Flow and Timing** | | | | Moderate | | | | | Moderate | | | | |  |
| **Overall Bias** | | Moderate | | | | High | | | |  |  |  |  |  |

# Automated Wound Tissue Segmentation Using DL - Ramachandram et al. (2022)

#### **1. Patient Selection**

- Dataset (465,000 images) spans various imaging conditions and skin tones, addressing bias concerns.
- **Risk of Bias:** Low
- **Applicability:** High

#### **2. Index Test and Reference Standard**

- Deep learning applied for tissue segmentation. Labels created by consensus from wound care clinicians.
- Inter- and intra-rater variability documented, improving reliability.
- **Risk of Bias:** Low
- **Applicability:** High

#### **3. Flow and Timing**

- Comprehensive image acquisition with no exclusions detailed.
- Temporal relationships between data points not addressed.
- **Risk of Bias:** Low
- **Applicability:** High

#### **4. Overall Bias and Applicability**

- **Bias:** Low
- **Applicability:** High

| **Domain** | **Risk of Bias** | | | | | **Applicability** | | | | | | | |  |
| --- | --- | --- | --- | --- | --- | --- | --- | --- | --- | --- | --- | --- | --- | --- |
| **Patient Selection** | | | Low | | | | High | | | |  |  |  |  |
| **Index Test/Ref Standard** | | | | | | | | | Low | | | High | | |
| **Flow and Timing** | | | | Low | | | | High | | | | |  |  |
| **Overall Bias** | | Low | | | High | | | | |  |  |  |  |  |

# Spectral Clustering for Wound Segmentation - Dhane et al. (2016)

#### **1. Patient Selection**

- Dataset (105 images) includes diverse wound types (pressure ulcers, diabetic foot ulcers, etc.) but is limited in size.
- **Risk of Bias:** Moderate
- **Applicability:** Moderate

#### **2. Index Test and Reference Standard**

- Spectral clustering and k-means applied, with results compared to ground truth labeled by dermatologists.
- Lack of blinding introduces potential bias.
- **Risk of Bias:** Moderate
- **Applicability:** High

#### **3. Flow and Timing**

- Limited follow-up data and no time-course evaluation.
- **Risk of Bias:** Moderate
- **Applicability:** Moderate

#### **4. Overall Bias and Applicability**

- **Bias:** Moderate
- **Applicability:** Moderate

| **Domain** | **Risk of Bias** | | | | **Applicability** | | | | | | |  |  |  |
| --- | --- | --- | --- | --- | --- | --- | --- | --- | --- | --- | --- | --- | --- | --- |
| **Patient Selection** | | | Moderate | | | | | Moderate | | | | |  |  |
| **Index Test/Ref Standard** | | | | | | | Moderate | | | | High | | | |
| **Flow and Timing** | | | | Moderate | | | | | Moderate | | | | |  |
| **Overall Bias** | | Moderate | | | | Moderate | | | |  |  |  |  |  |

# Predicting Wound Healing Time with ML - Berezo et al. (2021)

#### **1. Patient Selection**

- Dataset derived from 1.2M wounds across multiple facilities, ensuring representativeness.
- **Risk of Bias:** Low
- **Applicability:** High

#### **2. Index Test and Reference Standard**

- Gradient-boosted decision trees predict healing time using EHR data, validated using SHAP.
- **Risk of Bias:** Low
- **Applicability:** High

#### **3. Flow and Timing**

- Models predict healing at multiple time points (4, 8, and 12 weeks), incorporating temporal assessment.
- **Risk of Bias:** Low
- **Applicability:** High

#### **4. Overall Bias and Applicability**

- **Bias:** Low
- **Applicability:** High

| **Domain** | **Risk of Bias** | | | | | **Applicability** | | | | | | | |  |
| --- | --- | --- | --- | --- | --- | --- | --- | --- | --- | --- | --- | --- | --- | --- |
| **Patient Selection** | | | Low | | | | High | | | |  |  |  |  |
| **Index Test/Ref Standard** | | | | | | | | | Low | | | High | | |
| **Flow and Timing** | | | | Low | | | | High | | | | |  |  |
| **Overall Bias** | | Low | | | High | | | | |  |  |  |  |  |

# Multi-modal wound classification using wound image and location by deep neural network

1. **Patient Selection:** Data were sourced from two datasets, AZH and Medetec, with some diversity in wound types. However, limited information on how representative these datasets are and whether patient selection followed rigorous inclusion/exclusion criteria raises concerns about bias.
2. **Index Test:** The multi-modal approach incorporating wound images and location adds novelty, but there is no mention of how wound labels were validated independently or through consensus.
3. **Flow and Timing:** Limited detail about temporal aspects or potential exclusions affects clarity.
4. **Recommendations:** Improve dataset diversity and detail inter-rater validation for labeling wounds.

| **Domain** | **Risk of Bias** | **Applicability** |
| --- | --- | --- |
| **Patient Selection** | Moderate | Moderate |
| **Index Test/Ref Standard** | Moderate | High |
| **Flow and Timing** | Moderate | Moderate |
| **Overall Bias** | Moderate | High |

# The Enlightening Role of Explainable Artificial Intelligence in Chronic Wound Classification

1. **Patient Selection:** The dataset included a range of chronic wound types, but patient selection was not described comprehensively.
2. **Index Test:** The use of explainable AI (XAI) is a significant strength. However, no mention of blinded validation introduces potential bias.
3. **Flow and Timing:** The study provides comprehensive technical details, but patient flow through the study and exclusion criteria are not emphasized.
4. **Recommendations:** Strengthen descriptions of patient inclusion and validation processes.

| **Domain** | **Risk of Bias** | **Applicability** |
| --- | --- | --- |
| **Patient Selection** | Moderate | High |
| **Index Test/Ref Standard** | Moderate | High |
| **Flow and Timing** | Low | High |
| **Overall Bias** | Moderate | High |

# Comparison of Hybrid Convolutional Neural Networks Models for Diabetic Foot Ulcer Classification

1. **Patient Selection:** The study uses a single dataset with augmentation but lacks detail about patient diversity.
2. **Index Test:** Hybrid convolutional networks showed high performance; however, ground truth validation for labels is not elaborated upon.
3. **Flow and Timing:** Exclusion details and temporal alignment between data acquisition and model testing are well explained.
4. **Recommendations:** Include external validation using an independent dataset.

| **Domain** | **Risk of Bias** | **Applicability** |
| --- | --- | --- |
| **Patient Selection** | Moderate | High |
| **Index Test/Ref Standard** | Moderate | High |
| **Flow and Timing** | Low | High |
| **Overall Bias** | Moderate | High |

# A Deep Learning Approach for Diabetic Foot Ulcer Classification and Recognition

1. **Patient Selection:** Utilized a well-curated dataset (DFU2020) and augmentation techniques, ensuring better representation.
2. **Index Test:** Pre-trained CNN architectures achieved impressive accuracy, but inter-rater reliability or validation is not mentioned.
3. **Flow and Timing:** Considers two binary classification tasks but lacks comprehensive temporal evaluation details.
4. **Recommendations:** Add inter-rater validation for dataset labels and validate results across independent cohorts.

| **Domain** | **Risk of Bias** | **Applicability** |
| --- | --- | --- |
| **Patient Selection** | Low | High |
| **Index Test/Ref Standard** | Moderate | High |
| **Flow and Timing** | Moderate | High |
| **Overall Bias** | Moderate | High |

# Fusion of Hand-crafted and Deep Features for Automatic Diabetic Foot Ulcer Classification

1. **Patient Selection:** Uses a mixed dataset, but details about diversity and representativeness are lacking.
2. **Index Test:** Fusion of hand-crafted and deep features showed strong performance, but validation procedures are unclear.
3. **Flow and Timing:** Reasonable handling of fusion vectors for classification, but time-course data inclusion is absent.
4. **Recommendations:** Incorporate diversity in datasets and detail inter-rater validation.

| **Domain** | **Risk of Bias** | **Applicability** |
| --- | --- | --- |
| **Patient Selection** | Moderate | Moderate |
| **Index Test/Ref Standard** | Moderate | High |
| **Flow and Timing** | Moderate | High |
| **Overall Bias** | Moderate | High |

# Skin Tear Classification Using Machine Learning - Nagata et al. (2021)

- 1. **Patient Selection:** The dataset consisted of 31 skin tear images, with a focus on specific wound types. The sample size is limited and may not fully represent the population of skin tears.
  2. **Index Test:** The classification was performed using Support Vector Machines (SVM) and Random Forest (RF). Validation was performed using leave-one-out cross-validation, but there is no mention of blinded assessments.
  3. **Flow and Timing:** No discussion on potential exclusions or the time frame between image acquisition and evaluation.
  4. **Recommendations:** Increase dataset diversity and clarify blinding procedures during reference standard labeling.

| **Domain** | **Risk of Bias** | **Applicability** |
| --- | --- | --- |
| **Patient Selection** | Moderate | Moderate |
| **Index Test/Ref Standard** | Moderate | High |
| **Flow and Timing** | Moderate | Moderate |
| **Overall Bias** | Moderate | High |

# Automatic Measurement of Pressure Ulcers Using SVM and GrabCut - Lara e Silva et al. (2021)

1. **Patient Selection:** The study analyzed 105 pressure ulcer images, which are clinically relevant but limited in scope.
2. **Index Test:** The use of SVM with a modified GrabCut approach showed strong results (96% accuracy). However, the reference standard (ground truth) lacks detail on validation methods.
3. **Flow and Timing:** The segmentation process and evaluation were well-defined, but additional details on exclusions were absent.
4. **Recommendations:** Broaden dataset size and detail inter-rater validation processes for ground truth images.

| **Domain** | **Risk of Bias** | **Applicability** |
| --- | --- | --- |
| **Patient Selection** | Moderate | Moderate |
| **Index Test/Ref Standard** | Moderate | High |
| **Flow and Timing** | Low | High |
| **Overall Bias** | Moderate | High |

# Automated Tissue Classification Framework for Chronic Wounds Mukherjee et al. (2014)

| **Domain** | **Risk of Bias** | **Applicability** |
| --- | --- | --- |
| **Patient Selection** | Low | High |
| **Index Test/Ref Standard** | Low | High |
| **Flow and Timing** | Low | High |
| **Overall Bias** | Low | High |

- **Qualitative Assessment**:
  - **Patient Selection:** The study used a representative dataset with chronic wound types. Segmentation protocols were validated by clinical experts.
  - **Index Test:** High-performing SVM-based tissue classification achieved strong accuracy for various wound tissue types.
  - **Flow and Timing:** A clear process of segmentation and classification with thorough validation.
  - **Recommendations:** Expand validation across independent datasets for generalizability.

# Semantic Segmentation of Smartphone Wound Images: AHRF vs. CNN - Wagh et al. (2020)

- 1. **Patient Selection:** The dataset (<300 images) limits statistical power for deep learning models but is clinically relevant.
  2. **Index Test:** Deep learning approaches (FCN, U-Net, DeepLabV3) performed better than AHRF, but no blinding was applied during reference standard creation.
  3. **Flow and Timing:** Temporal and methodological details are well-articulated.
  4. **Recommendations:** Validate across larger datasets and ensure independent ground truth validation.

| **Domain** | **Risk of Bias** | **Applicability** |
| --- | --- | --- |
| **Patient Selection** | Moderate | High |
| **Index Test/Ref Standard** | Moderate | High |
| **Flow and Timing** | Low | High |
| **Overall Bias** | Moderate | High |

# Fuzzy Spectral Clustering for Chronic Wounds - Dhane et al. (2017)

- 1. **Patient Selection:** Dataset included 70 wound images but lacked diversity.
  2. **Index Test:** The fuzzy spectral clustering approach demonstrated robustness but lacked clarity on blinded validation.
  3. **Flow and Timing:** Limited information on dataset exclusions or the timing of data acquisition.
  4. **Recommendations:** Expand dataset diversity and incorporate blinded ground truth validation.

| **Domain** | **Risk of Bias** | **Applicability** |
| --- | --- | --- |
| **Patient Selection** | Moderate | Moderate |
| **Index Test/Ref Standard** | Moderate | High |
| **Flow and Timing** | Moderate | Moderate |
| **Overall Bias** | Moderate | Moderate |

# Multi-view Data Augmentation to Improve Wound Segmentation

1. **Patient Selection:** The dataset of 270 images provided a diverse representation of chronic wounds.
2. **Index Test:** The approach used multiple camera angles and best-view selection, but ground truth validation was not independently verified.
3. **Flow and Timing:** Time between acquisition and validation was not reported, affecting temporal reliability.
4. **Recommendations:** Include independent validation of wound labels.

| **Domain** | **Risk of Bias** | **Applicability** |
| --- | --- | --- |
| **Patient Selection** | Low | High |
| **Index Test/Ref Standard** | Moderate | High |
| **Flow and Timing** | Moderate | High |
| **Overall Bias** | Moderate | High |

# EfficientNet Models for Diabetic Foot Ulcer Infection and Ischemia Classification

1. **Patient Selection:** Utilized the DFUC2021 dataset, a high-quality resource with clearly defined labels.
2. **Index Test:** EfficientNet achieved high classification performance, with rigorous cross-validation.
3. **Flow and Timing:** Comprehensive methodological clarity ensured robustness.
4. **Recommendations:** Incorporate additional datasets for external validation.

| **Domain** | **Risk of Bias** | **Applicability** |
| --- | --- | --- |
| **Patient Selection** | Low | High |
| **Index Test/Ref Standard** | Low | High |
| **Flow and Timing** | Low | High |
| **Overall Bias** | Low | High |

# Segmentation of Chronic Wound Areas Using Clustering Techniques

1. **Patient Selection:** Relied on 77 images from the Medetec database, which may limit generalizability.
2. **Index Test:** K-means and fuzzy c-means clustering in the YDbDr color space showed good performance but lacked detailed validation.
3. **Flow and Timing:** Minimal exclusions and clear segmentation steps improve reliability.
4. **Recommendations:** Use larger, more diverse datasets and standardize validation protocols.

| **Domain** | **Risk of Bias** | **Applicability** |
| --- | --- | --- |
| **Patient Selection** | Moderate | High |
| **Index Test/Ref Standard** | Moderate | High |
| **Flow and Timing** | Low | High |
| **Overall Bias** | Moderate | High |

# Smartphone and Tablet Photography for Diabetic Foot Ulcer Healing Prediction

1. **Patient Selection:** Included 208 wounds with a clear definition of healed vs. non-healed outcomes.
2. **Index Test:** Employed both handcrafted and deep learning features but lacked independent validation for wound segmentation.
3. **Flow and Timing:** Well-documented with rigorous cross-validation procedures.
4. **Recommendations:** Validate imaging biomarkers across independent datasets.

| **Domain** | **Risk of Bias** | **Applicability** |
| --- | --- | --- |
| **Patient Selection** | Low | High |
| **Index Test/Ref Standard** | Moderate | High |
| **Flow and Timing** | Low | High |
| **Overall Bias** | Moderate | High |

# Semi-Supervised Learning in Automated Wound Segmentation

1. **Patient Selection:** Utilized a large dataset (Deepskin) of 1564 images, which is highly representative.
2. **Index Test:** U-Net architecture demonstrated strong segmentation performance but lacked blinded validation.
3. **Flow and Timing:** Transparent and iterative semi-supervised learning improved robustness.
4. **Recommendations:** Use additional datasets to test generalization.

| **Domain** | **Risk of Bias** | **Applicability** |
| --- | --- | --- |
| **Patient Selection** | Low | High |
| **Index Test/Ref Standard** | Moderate | High |
| **Flow and Timing** | Low | High |
| **Overall Bias** | Moderate | High |

# Chronic Wound Assessment and Infection Detection Method

- 1. **Patient Selection:** Included 293 wound images captured under diverse conditions, reflecting real-world variability.
  2. **Index Test:** Edge-based segmentation and SVM-based infection detection showed good performance but lacked blinded validation.
  3. **Flow and Timing:** Clear methodology but with minimal discussion on potential exclusions.
  4. **Recommendations:** Incorporate larger datasets and clarify validation protocols.

| **Domain** | **Risk of Bias** | **Applicability** |
| --- | --- | --- |
| **Patient Selection** | Low | High |
| **Index Test/Ref Standard** | Moderate | High |
| **Flow and Timing** | Moderate | High |
| **Overall Bias** | Moderate | High |

# A Unified Framework for Wound Infection Detection

- 1. **Patient Selection:** Dataset (480 images) lacked diversity, with all samples collected from a single center.
  2. **Index Test:** AUC-based evaluation showed superiority over traditional classifiers, but labeling lacked blinding.
  3. **Flow and Timing:** Robust validation but no details on exclusions or time intervals.
  4. **Recommendations:** Broaden dataset sources and validate labeling procedures.

| **Domain** | **Risk of Bias** | **Applicability** |
| --- | --- | --- |
| **Patient Selection** | Moderate | High |
| **Index Test/Ref Standard** | Moderate | High |
| **Flow and Timing** | Moderate | High |
| **Overall Bias** | Moderate | High |

# Recognition of Ischaemia and Infection in DFU

- 1. **Patient Selection:** Images lacked standardization in terms of metadata like age, sex, and lighting conditions.
  2. **Index Test:** Ensemble CNN models achieved robust performance, with validated labels by two experts.
  3. **Flow and Timing:** Exclusions not justified, and no timing discussion between image capture and ground truth validation.
  4. **Recommendations:** Standardize metadata and improve exclusion criteria documentation.

| **Domain** | **Risk of Bias** | **Applicability** |
| --- | --- | --- |
| **Patient Selection** | Moderate | High |
| **Index Test/Ref Standard** | Low | High |
| **Flow and Timing** | Moderate | High |
| **Overall Bias** | Moderate | High |

# Wound Segmentation Network with Location Information

- 1. **Patient Selection:** Labeled dataset (950 images) with sufficient variety for segmentation tasks.
  2. **Index Test:** Location-enhanced CNN provided superior segmentation accuracy.
  3. **Flow and Timing:** Lack of clarity on how exclusions were handled.
  4. **Recommendations:** Include more metadata on patient demographics and explicit exclusion criteria.

| **Domain** | **Risk of Bias** | **Applicability** |
| --- | --- | --- |
| **Patient Selection** | Moderate | High |
| **Index Test/Ref Standard** | Low | High |
| **Flow and Timing** | Moderate | High |
| **Overall Bias** | Moderate | High |

# Superpixel-Driven Deep Learning for Wound Analysis

- 1. **Patient Selection:** Large dataset (179,572 superpixels) with diverse dermatological conditions.
  2. **Index Test:** QTDU framework with ResNet-based model achieved high sensitivity and specificity.
  3. **Flow and Timing:** Comprehensive details provided for all included cases.
  4. **Recommendations:** None, as the study exhibits high methodological rigor.

| **Domain** | **Risk of Bias** | **Applicability** |
| --- | --- | --- |
| **Patient Selection** | Low | High |
| **Index Test/Ref Standard** | Low | High |
| **Flow and Timing** | Low | High |
| **Overall Bias** | Low | High |

# ASURA Framework for Skin Ulcer Measurement

1. **Patient Selection:** The study used diverse datasets with controlled conditions for image capture.
2. **Index Test:** ASURA demonstrated high precision in segmentation and measurement tasks, with validated results against human-annotated data.
3. **Flow and Timing:** Detailed steps for data processing were included, but the time intervals between measurements and evaluations were unclear.
4. **Recommendations:** Add temporal data to better analyze segmentation consistency over time.

| **Domain** | **Risk of Bias** | **Applicability** |
| --- | --- | --- |
| **Patient Selection** | Low | High |
| **Index Test/Ref Standard** | Low | High |
| **Flow and Timing** | Moderate | High |
| **Overall Bias** | Low | High |

# CNN-Based Wound Detection with Pretrained Architectures

1. **Patient Selection:** The dataset was based on sacral pressure ulcers, limiting generalizability for diabetic wounds or other wound types.
2. **Index Test:** While U-Net architectures showed robust performance, no blinded validation of segmentations was conducted.
3. **Flow and Timing:** Clear temporal validation and testing procedures enhance reliability.
4. **Recommendations:** Expand datasets and consider blinding for segmentation validation.

| **Domain** | **Risk of Bias** | **Applicability** |
| --- | --- | --- |
| **Patient Selection** | Moderate | High |
| **Index Test/Ref Standard** | Moderate | High |
| **Flow and Timing** | Low | High |
| **Overall Bias** | Moderate | High |

# Fine-Grained Diabetic Wound Depth and Granulation Tissue Amount Assessment Using Bilinear Convolutional Neural Network

# **Patient Selection:** The dataset was well-labeled but relatively small for a five-class fine-grained classification task.

1. **Index Test:** Bi-CNN demonstrated superior performance compared to traditional CNNs, with robust validation results.
2. **Flow and Timing:** No explicit mention of the time intervals between imaging and classification evaluations.
3. **Recommendations:** Increase the dataset size and report temporal data for robustness.

| **Domain** | **Risk of Bias** | **Applicability** |
| --- | --- | --- |
| **Patient Selection** | Moderate | High |
| **Index Test/Ref Standard** | Low | High |
| **Flow and Timing** | Moderate | High |
| **Overall Bias** | Moderate | High |

# ﻿Real-time classification on oral ulcer images with residual network and image enhancement

1. **Patient Selection:** Limited oral ulcer dataset affects model generalizability.
2. **Index Test:** Residual networks with transfer learning achieved high accuracy, but dataset limitations remain a concern.
3. **Flow and Timing:** The flow of data was clearly documented, with robust testing procedures.
4. **Recommendations:** Broaden the dataset and validate results across independent test samples.

| **Domain** | **Risk of Bias** | **Applicability** |
| --- | --- | --- |
| **Patient Selection** | Moderate | High |
| **Index Test/Ref Standard** | Low | High |
| **Flow and Timing** | Low | High |
| **Overall Bias** | Moderate | High |

# AI-Assisted Assessment of Wound Tissue with Automatic Color and Measurement Calibration on Images Taken with a Smartphone

1. **Patient Selection:** Dataset diversity was ensured, with annotations from experienced clinicians.
2. **Index Test:** AI-based segmentation with color calibration provided robust results, showing strong agreement with human annotations.
3. **Flow and Timing:** Comprehensive testing and validation procedures were reported.
4. **Recommendations:** Expand datasets for broader generalizability.

| **Domain** | **Risk of Bias** | **Applicability** |
| --- | --- | --- |
| **Patient Selection** | Low | High |
| **Index Test/Ref Standard** | Low | High |
| **Flow and Timing** | Low | High |
| **Overall Bias** | Low | High |

**Qualitative Assessment**:

# The Random Forest Model Has the Best Accuracy Among the Four Pressure Ulcer Prediction Models Using Machine Learning Algorithms

1. **Patient Selection:** Large and diverse dataset of 5814 patients ensures robustness in predictions.
2. **Index Test:** Various ML algorithms were tested, but cross-validation details were minimal.
3. **Flow and Timing:** The exclusion criteria were clear, but limited details on time intervals affect temporal reliability.
4. **Recommendations:** Include more granular validation steps for each model's predictive capacity.

| **Domain** | **Risk of Bias** | **Applicability** |
| --- | --- | --- |
| **Patient Selection** | Low | High |
| **Index Test/Ref Standard** | Moderate | High |
| **Flow and Timing** | Moderate | High |
| **Overall Bias** | Moderate | High |

# FusionSegNet: Fusing global foot features and local wound features to diagnose diabetic foot

1. **Patient Selection:** Utilized 1211 images from a well-curated dataset, but limited diversity across patient demographics.
2. **Index Test:** FusionSegNet showed high AUC and F1 scores, reflecting strong performance.
3. **Flow and Timing:** Sequential and logical flow of testing data ensures reliability.
4. **Recommendations:** Validate model performance on external datasets for generalizability.

| **Domain** | **Risk of Bias** | **Applicability** |
| --- | --- | --- |
| **Patient Selection** | Moderate | High |
| **Index Test/Ref Standard** | Low | High |
| **Flow and Timing** | Low | High |
| **Overall Bias** | Low | High |

# Multiclass wound image classification using an ensemble deep CNN-based classifier

1. **Patient Selection:** The dataset included 538 images spanning multiple wound types, but lacked sufficient metadata on sample acquisition.
2. **Index Test:** Ensemble CNN demonstrated robust classification, with accuracy up to 96.4%.
3. **Flow and Timing:** Clear documentation of steps improves replicability.
4. **Recommendations:** Add details on image acquisition protocols to enhance replicability.

| **Domain** | **Risk of Bias** | **Applicability** |
| --- | --- | --- |
| **Patient Selection** | Moderate | High |
| **Index Test/Ref Standard** | Low | High |
| **Flow and Timing** | Low | High |
| **Overall Bias** | Low | High |

# Mobile App for Wound Localization Using Deep Learning

1. **Patient Selection:** Dataset size and diversity were adequate but could benefit from multi-center sources.
2. **Index Test:** YOLOv3 provided superior performance with mAP 97.3%, ensuring high reliability.
3. **Flow and Timing:** Comprehensive flow with robust data preprocessing and testing.
4. **Recommendations:** Integrate performance validation across broader clinical settings.

| **Domain** | **Risk of Bias** | **Applicability** |
| --- | --- | --- |
| **Patient Selection** | Moderate | High |
| **Index Test/Ref Standard** | Low | High |
| **Flow and Timing** | Low | High |
| **Overall Bias** | Low | High |

# Wound Intensity Correction and Segmentation with CNN

1. **Patient Selection:** Limited sample diversity affects generalizability.
2. **Index Test:** Innovative CNN-based segmentation improves accuracy in intensity-corrected images.
3. **Flow and Timing:** Insufficient information on timing between image acquisition and correction steps.
4. **Recommendations:** Use larger, multi-center datasets and include temporal data for reliability.

| **Domain** | **Risk of Bias** | **Applicability** |
| --- | --- | --- |
| **Patient Selection** | Moderate | High |
| **Index Test/Ref Standard** | Low | High |
| **Flow and Timing** | Moderate | High |
| **Overall Bias** | Moderate | High |

# DFUNet: Convolutional Neural Networks for Diabetic Foot Ulcer Classification

1. **Patient Selection:** Large dataset of DFU and healthy skin images, ensuring representation of real-world scenarios.
2. **Index Test:** DFUNet's AUC of 0.961 reflects strong discriminatory ability, with sufficient validation through 10-fold cross-validation.
3. **Flow and Timing:** Detailed description of dataset collection and annotation protocols enhances study robustness.
4. **Recommendations:** Include more demographic diversity in datasets for improved generalizability.

| **Domain** | **Risk of Bias** | **Applicability** |
| --- | --- | --- |
| **Patient Selection** | Low | High |
| **Index Test/Ref Standard** | Low | High |
| **Flow and Timing** | Low | High |
| **Overall Bias** | Low | High |

# Experimental Study on Wound Area Measurement with Mobile Devices

1. **Patient Selection:** Limited sample diversity and reliance on a small dataset affect reliability.
2. **Index Test:** Use of OpenCV for wound area segmentation is promising but lacks blinded validation.
3. **Flow and Timing:** Insufficient clarity on the time intervals and exclusions.
4. **Recommendations:** Incorporate larger datasets with diverse imaging conditions and ensure more rigorous validation.

| **Domain** | **Risk of Bias** | **Applicability** |
| --- | --- | --- |
| **Patient Selection** | Moderate | High |
| **Index Test/Ref Standard** | Moderate | High |
| **Flow and Timing** | Moderate | High |
| **Overall Bias** | Moderate | High |

# DFU_QUTNet: Classification Using Deep Convolutional Neural Network

1. **Patient Selection:** Comprehensive dataset of 754-foot images ensures sufficient diversity.
2. **Index Test:** DFU_QUTNet outperformed existing models with an F1-score of 94.5%, showcasing strong predictive power.
3. **Flow and Timing:** Clear methodology with appropriate validation enhances robustness.
4. **Recommendations:** Extend validation to external datasets for broader clinical utility.

| **Domain** | **Risk of Bias** | **Applicability** |
| --- | --- | --- |
| **Patient Selection** | Low | High |
| **Index Test/Ref Standard** | Low | High |
| **Flow and Timing** | Low | High |
| **Overall Bias** | Low | High |

# Granulation Tissue Detection Model for Chronic Wound Healing

1. **Patient Selection:** Dataset with 219 wound images from 100 DM patients lacks significant diversity.
2. **Index Test:** ResNet-based segmentation provides accurate granulation detection, with IOU scores above 0.5.
3. **Flow and Timing:** Minimal discussion on exclusions or image acquisition timing.
4. **Recommendations:** Expand the dataset size and include more detailed temporal data.

| **Domain** | **Risk of Bias** | **Applicability** |
| --- | --- | --- |
| **Patient Selection** | Moderate | High |
| **Index Test/Ref Standard** | Low | High |
| **Flow and Timing** | Moderate | High |
| **Overall Bias** | Moderate | High |

# Classification of Diabetic Foot Ulcers Using Class Knowledge Banks

1. **Patient Selection:** Comprehensive dataset with attention to class imbalance ensures robust testing conditions.
2. **Index Test:** Class Knowledge Banks (CKB) significantly enhance classification accuracy and balance.
3. **Flow and Timing:** Clear protocol ensures replicability and reliability.
4. **Recommendations:** Explore additional medical imaging modalities to improve detection versatility.

| **Domain** | **Risk of Bias** | **Applicability** |
| --- | --- | --- |
| **Patient Selection** | Low | High |
| **Index Test/Ref Standard** | Low | High |
| **Flow and Timing** | Low | High |
| **Overall Bias** | Low | High |

# Validation of AI Medical Device for Wound Assessment

1. **Patient Selection:** The dataset included a diverse patient population, but inclusion criteria lacked detail on demographic stratification.
2. **Index Test:** Validation of AI-powered wound assessment devices showed high agreement with physicians (97%), indicating robust performance.
3. **Flow and Timing:** Time intervals between assessments and physician evaluations were not clearly documented.
4. **Recommendations:** Include explicit details on timing and external validation datasets to improve generalizability.

| **Domain** | **Risk of Bias** | **Applicability** |
| --- | --- | --- |
| **Patient Selection** | Moderate | High |
| **Index Test/Ref Standard** | Low | High |
| **Flow and Timing** | Moderate | High |
| **Overall Bias** | Moderate | High |

# Boundary Determination for Foot Ulcers with AHRF Framework

1. **Patient Selection:** Mixed datasets with controlled and uncontrolled image acquisition were included, but demographic details were limited.
2. **Index Test:** AHRF framework improved wound boundary determination, achieving specificity >95%.
3. **Flow and Timing:** Sequential data processing was well-documented.
4. **Recommendations:** Broaden patient demographics and validate results with independent datasets.

| **Domain** | **Risk of Bias** | **Applicability** |
| --- | --- | --- |
| **Patient Selection** | Moderate | High |
| **Index Test/Ref Standard** | Moderate | High |
| **Flow and Timing** | Low | High |
| **Overall Bias** | Moderate | High |

# Transfer Learning for DFU Classification

1. **Patient Selection:** The dataset was small and lacked ethnic diversity, limiting generalizability.
2. **Index Test:** Transfer learning achieved high F1 scores (97.6%) when using domain-specific pre-training.
3. **Flow and Timing:** Training and validation processes were clearly described, but testing on external datasets was absent.
4. **Recommendations:** Expand datasets and validate model on diverse clinical populations.

| **Domain** | **Risk of Bias** | **Applicability** |
| --- | --- | --- |
| **Patient Selection** | Moderate | High |
| **Index Test/Ref Standard** | Low | High |
| **Flow and Timing** | Moderate | High |
| **Overall Bias** | Moderate | High |

# ML Models for Hard-to-Heal DFUs

1. **Patient Selection:** Included a comprehensive dataset of 362 patients with well-documented predictors.
2. **Index Test:** Naïve Bayes model outperformed others, with AUC 0.864, demonstrating strong discriminatory power.
3. **Flow and Timing:** Clear methodology and robust data partitioning enhance reliability.
4. **Recommendations:** Further validation in external cohorts would strengthen findings.

| **Domain** | **Risk of Bias** | **Applicability** |
| --- | --- | --- |
| **Patient Selection** | Low | High |
| **Index Test/Ref Standard** | Low | High |
| **Flow and Timing** | Low | High |
| **Overall Bias** | Low | High |

# Predictive Models for Delayed Wound Healing

1. **Patient Selection:** Large dataset of over 59,000 patients across 68 centers enhances generalizability.
2. **Index Test:** Achieved AUC 0.842, reflecting strong predictive accuracy for delayed healing.
3. **Flow and Timing:** Sequential patient monitoring and exclusion criteria were well-documented.
4. **Recommendations:** Validate on datasets from other healthcare settings to confirm generalizability.

| **Domain** | **Risk of Bias** | **Applicability** |
| --- | --- | --- |
| **Patient Selection** | Low | High |
| **Index Test/Ref Standard** | Low | High |
| **Flow and Timing** | Low | High |
| **Overall Bias** | Low | High |

# Constructing Inpatient Pressure Injury Prediction Models Using Machine Learning Techniques

1. **Patient Selection:** The dataset of 11,838 records was robust but restricted to a single hospital, limiting external generalizability.
2. **Index Test:** The study used decision tree, logistic regression, and random forest models effectively to identify critical risk factors.
3. **Flow and Timing:** Minimal detail on exclusions and missing data handling could introduce minor biases.
4. **Recommendations:** Validation across diverse healthcare systems is needed to improve generalizability.

| **Domain** | **Risk of Bias** | **Applicability** |
| --- | --- | --- |
| **Patient Selection** | Low | Moderate |
| **Index Test/Ref Standard** | Low | High |
| **Flow and Timing** | Moderate | High |
| **Overall Bias** | Low | High |

# A Prognostic Model of Surgical Site Infection Using Daily Clinical Wound Assessment

1. **Patient Selection:** Prospective cohort of 851 patients is comprehensive but lacks global demographic diversity.
2. **Index Test:** Naïve Bayes classifier with strong AUC values (0.76) provides reliable predictions.
3. **Flow and Timing:** Exclusions due to insufficient observations raise concerns about potential bias.
4. **Recommendations:** Include more diverse datasets and report missing data handling procedures explicitly.

| **Domain** | **Risk of Bias** | **Applicability** |
| --- | --- | --- |
| **Patient Selection** | Moderate | High |
| **Index Test/Ref Standard** | Low | High |
| **Flow and Timing** | Moderate | High |
| **Overall Bias** | Moderate | High |

# Surgical Wounds Assessment System for Self-Care

1. **Patient Selection:** Images from non-professional cameras were analyzed, but the dataset size and quality variability raise concerns.
2. **Index Test:** Machine learning combined with image segmentation techniques achieved >90% accuracy in state assessment.
3. **Flow and Timing:** Limited detail on how variability in image quality was addressed could affect reliability.
4. **Recommendations:** Expand the dataset and integrate higher-resolution imaging for enhanced validation.

| **Domain** | **Risk of Bias** | **Applicability** |
| --- | --- | --- |
| **Patient Selection** | Moderate | High |
| **Index Test/Ref Standard** | Low | High |
| **Flow and Timing** | Moderate | High |
| **Overall Bias** | Moderate | High |

# Machine Learning-Based Pressure Ulcer Prediction in Modular Critical Care Data

1. **Patient Selection:** Leveraged the extensive MIMIC-IV database with robust feature engineering and classification methods.
2. **Index Test:** Random forest achieved 96% accuracy, reflecting high performance for PU predictions.
3. **Flow and Timing:** Comprehensive feature selection and analysis enhance reliability.
4. **Recommendations:** Validate results with external datasets and evaluate interpretability for clinical adoption.

| **Domain** | **Risk of Bias** | **Applicability** |
| --- | --- | --- |
| **Patient Selection** | Low | High |
| **Index Test/Ref Standard** | Low | High |
| **Flow and Timing** | Low | High |
| **Overall Bias** | Low | High |

# Quantifying Digital Ulcers in Systemic Sclerosis Using Digital Planimetry

1. **Patient Selection:** Comprehensive data from 107 digital ulcers in 36 patients provide strong reliability metrics.
2. **Index Test:** Planimetry methods (ellipse and free-hand ROI) demonstrated >95% intra-rater reliability.
3. **Flow and Timing:** Missing details on patient follow-up intervals could impact longitudinal assessments.
4. **Recommendations:** Implement national guidelines for DU measurement and expand the tool to larger populations.

| **Domain** | **Risk of Bias** | **Applicability** |
| --- | --- | --- |
| **Patient Selection** | Low | High |
| **Index Test/Ref Standard** | Low | High |
| **Flow and Timing** | Moderate | High |
| **Overall Bias** | Low | High |

# Clinical Validation of an AI-Enabled Wound Imaging Mobile Application in Diabetic Foot Ulcers

1. **Patient Selection:** Robust dataset with clear inclusion/exclusion criteria focusing on diabetic foot ulcers (DFUs).
2. **Index Test:** Excellent intra- and inter-rater reliability of AI measurements against traditional manual methods.
3. **Flow and Timing:** Consistent testing and validation timelines support reliability.
4. **Recommendations:** Expand validation to include external datasets for broader generalizability.

| **Domain** | **Risk of Bias** | **Applicability** |
| --- | --- | --- |
| **Patient Selection** | Low | High |
| **Index Test/Ref Standard** | Low | High |
| **Flow and Timing** | Low | High |
| **Overall Bias** | Low | High |

# Computer-Assisted Differential Diagnosis of Pyoderma Gangrenosum and Venous Ulcers Using Deep Neural Networks

1. **Patient Selection:** Dataset is small and lacks detailed demographic diversity.
2. **Index Test:** CNN performed well but lacked external validation to generalize beyond the study population.
3. **Flow and Timing:** Sequential analysis is solid, but insufficient details on exclusions and retesting protocols.
4. **Recommendations:** Validate CNN against a larger, diverse dataset.

| **Domain** | **Risk of Bias** | **Applicability** |
| --- | --- | --- |
| **Patient Selection** | Moderate | High |
| **Index Test/Ref Standard** | Moderate | High |
| **Flow and Timing** | Moderate | High |
| **Overall Bias** | Moderate | High |

# Decision Tree Analysis of Pressure Ulcer Risk Factors in Long-Term Care Facilities

1. **Patient Selection:** Dataset appears comprehensive but lacks geographic diversity.
2. **Index Test:** Decision tree achieved strong accuracy but required more comprehensive feature validation.
3. **Flow and Timing:** Limited detail on the timing of data collection.
4. **Recommendations:** Further detail on feature engineering and external validation required.

| **Domain** | **Risk of Bias** | **Applicability** |
| --- | --- | --- |
| **Patient Selection** | Moderate | Moderate |
| **Index Test/Ref Standard** | Moderate | Moderate |
| **Flow and Timing** | Moderate | Moderate |
| **Overall Bias** | Moderate | Moderate |

# Towards an AI-Based Objective Prognostic Model for Quantifying Wound Healing

1. **Patient Selection:** Large dataset provides solid representativeness.
2. **Index Test:** Deep learning achieved notable improvement over traditional methods like PUSH and BWAT.
3. **Flow and Timing:** No clear reporting on patient follow-ups or exclusions.
4. **Recommendations:** Broaden validation and provide longitudinal analysis of wound healing trajectories.

| **Domain** | **Risk of Bias** | **Applicability** |
| --- | --- | --- |
| **Patient Selection** | Low | High |
| **Index Test/Ref Standard** | Low | High |
| **Flow and Timing** | Moderate | High |
| **Overall Bias** | Low | High |

# Chronic Wound Image Augmentation and Assessment Using Semi-Supervised Progressive Multi-Granularity EfficientNet

1. **Patient Selection:** Inclusion of small labeled datasets (1639 images) augmented using semi-supervised learning. Limited diversity might affect generalizability.
2. **Index Test:** EfficientNet outperformed prior models in wound grading accuracy but lacked external validation.
3. **Flow and Timing:** Limited reporting on the progression of data augmentation and segmentation steps.
4. **Recommendations:** Conduct external validation across more diverse populations and detail dataset augmentation processes.

| **Domain** | **Risk of Bias** | **Applicability** |
| --- | --- | --- |
| **Patient Selection** | Moderate | High |
| **Index Test/Ref Standard** | Low | High |
| **Flow and Timing** | Moderate | High |
| **Overall Bias** | Moderate | High |

# Comprehensive Assessment of Fine-Grained Wound Images Using a Patch-Based CNN With Context-Preserving Attention

1. **Patient Selection**: Well-defined dataset with 1639 images annotated using the PWAT tool, covering multiple wound types.
2. **Index Test:** Achieved >80% accuracy and F1 scores using a DenseNet CNN architecture with context-preserving mechanisms.
3. **Flow and Timing:** No information on follow-up or longitudinal validation could introduce biases in clinical applications.
4. **Recommendations:** Include a longitudinal validation phase and extend analysis to external datasets for comprehensive testing.

| **Domain** | **Risk of Bias** | **Applicability** |
| --- | --- | --- |
| **Patient Selection** | Low | High |
| **Index Test/Ref Standard** | Low | High |
| **Flow and Timing** | Moderate | High |
| **Overall Bias** | Low | High |

# Automatic Segmentation and Measurement of Pressure Injuries Using Deep Learning Models and a LiDAR Camera

1. **Patient Selection**: Robust data collection from 528 patient images for segmentation and validation purposes.
2. **Index Test:** U-Net model outperformed Mask R-CNN for wound segmentation with a Dice coefficient of 0.8448.
3. **Flow and Timing:** Prospective validation was limited to one clinical setting, which may impact generalizability.
4. **Recommendations:** Validate the integration of LiDAR technology in broader settings with multiple clinical sites.

| **Domain** | **Risk of Bias** | **Applicability** |
| --- | --- | --- |
| **Patient Selection** | Low | High |
| **Index Test/Ref Standard** | Low | High |
| **Flow and Timing** | Moderate | High |
| **Overall Bias** | Low | High |

# YOLO-Based Deep Learning Model for Pressure Ulcer Detection and Classification

1. **Patient Selection:** Limited to specific hospital settings, potentially restricting generalizability.
2. **Index Test:** YOLOv5 outperformed traditional CNNs with higher mAP.
3. **Flow and Timing:** Insufficient details on validation timelines and exclusion criteria.
4. **Recommendations:** Validate the model with a larger, more varied dataset and report exclusion criteria in detail.

| **Domain** | **Risk of Bias** | **Applicability** |
| --- | --- | --- |
| **Patient Selection** | Moderate | High |
| **Index Test/Ref Standard** | Low | High |
| **Flow and Timing** | Moderate | High |
| **Overall Bias** | Moderate | High |

# Development of a Deep Learning-Based Tool to Assist Wound Classification

1. **Patient Selection**: Data collection from 2149 wound images was well-structured but lacked global demographic diversity.
2. **Index Test:** Multi-task CNN model successfully classified five wound types with performance comparable to specialists.
3. **Flow and Timing:** Absence of external validation and follow-up testing phases limit its robustness.
4. **Recommendations:** Broaden demographic representation in datasets and conduct external validations.

| **Domain** | **Risk of Bias** | **Applicability** |
| --- | --- | --- |
| **Patient Selection** | Moderate | High |
| **Index Test/Ref Standard** | Low | High |
| **Flow and Timing** | Moderate | High |
| **Overall Bias** | Moderate | High |

# Construction and Validation of an Image Discrimination Algorithm to Discriminate Necrosis from Wounds in Pressure Ulcers

1. **Patient Selection**: Small dataset (50 images) could introduce biases in algorithm development and validation.
2. **Index Test:** Algorithm achieved 100% concordance with expert evaluations but was not validated externally.
3. **Flow and Timing**: Narrow inclusion criteria and lack of longitudinal testing limit generalizability.
4. **Recommendations**: Expand dataset size and diversity; implement longitudinal studies for robust validation.

| **Domain** | **Risk of Bias** | **Applicability** |
| --- | --- | --- |
| **Patient Selection** | Moderate | Moderate |
| **Index Test/Ref Standard** | Moderate | Moderate |
| **Flow and Timing** | Moderate | Moderate |
| **Overall Bias** | Moderate | Moderate |

# Image Analysis System for Early Detection of Cardiothoracic Surgery Wound Alterations Using AI

1. Patient Selection: Data from 34 patients were included, but the imbalanced dataset (10.7% showing alterations) could introduce biases.
2. Index Test: Clear description of MobileNet-Unet for segmentation and ML models (SVM, RF) for classification ensures reliability.
3. Flow and Timing: Limited explanation of follow-up duration and potential patient exclusions.
4. Recommendations: Broaden dataset representation and provide comprehensive follow-up data.

| **Domain** | **Risk of Bias** | **Applicability** |
| --- | --- | --- |
| **Patient Selection** | Moderate | High |
| **Index Test/Ref Standard** | Low | High |
| **Flow and Timing** | Moderate | High |
| **Overall Bias** | Moderate | High |

# Simultaneous Segmentation and Classification of Pressure Injury Image Data Using Mask-R-CNN

1. **Patient Selection**: Dataset (969 images) well-defined, covering stages 1-4 of pressure injuries.
2. **Index Test:** Mask-R-CNN implementation rigorously validated with Dice coefficients and F1 scores.
3. **Flow and Timing**: Detailed explanation of training, validation, and test set split supports reproducibility.
4. **Recommendations**: Expand use cases beyond pressure injury for broader clinical applicability.

| **Domain** | **Risk of Bias** | **Applicability** |
| --- | --- | --- |
| **Patient Selection** | Low | High |
| **Index Test/Ref Standard** | Low | High |
| **Flow and Timing** | Low | High |
| **Overall Bias** | Low | High |

# Clinical Validation of AI Algorithms for Wound Measurement and Tissue Classification

1. **Patient Selection**: Limited dataset may not represent diverse patient populations.
2. **Index Test:** ResNet50 achieved strong results for tissue classification, validating its accuracy.
3. **Flow and Timing**: Absence of detailed longitudinal follow-up raises concerns about long-term reliability.
4. **Recommendations:** Conduct external validation and document dataset diversity.

| **Domain** | **Risk of Bias** | **Applicability** |
| --- | --- | --- |
| **Patient Selection** | Moderate | High |
| **Index Test/Ref Standard** | Low | High |
| **Flow and Timing** | Moderate | High |
| **Overall Bias** | Moderate | High |

# Pressure Ulcers Assessment System Using CNN for Diagnosis and Decision Making

1. **Patient Selection:** Retrospective study with clearly labeled dataset (327 images).
2. **Index Test:** Inception-ResNet-v2 used for erythema and necrotic tissue classification shows robust accuracy (97%-98.5%).
3. **Flow and Timing**: Well-documented timeline for data collection and analysis supports reproducibility.
4. **Recommendations**: Incorporate more real-world scenarios for wider applicability.

| **Domain** | **Risk of Bias** | **Applicability** |
| --- | --- | --- |
| **Patient Selection** | Low | High |
| **Index Test/Ref Standard** | Low | High |
| **Flow and Timing** | Low | High |
| **Overall Bias** | Low | High |

# Identifying Optimal Threshold for Image Segmentation Using PSO for Chronic Wound Assessment

1. **Patient Selection**: Dataset (50 images) is small and lacks comprehensive representation.
2. **Index Test**: Combination of Otsu's method with PSO improved segmentation quality in 46% of cases.
3. **Flow and Timing**: Sparse details about exclusions and validation timelines.
4. **Recommendations**: Increase dataset size and diversity; explore hybrid techniques for broader application.

| **Domain** | **Risk of Bias** | **Applicability** |
| --- | --- | --- |
| **Patient Selection** | Moderate | Moderate |
| **Index Test/Ref Standard** | Moderate | Moderate |
| **Flow and Timing** | Moderate | Moderate |
| **Overall Bias** | Moderate | Moderate |

# A Time Motion Study of Manual Versus Artificial Intelligence Methods for Wound Assessment

1. **Patient Selection**: Limited diversity in patient demographics restricts generalizability.
2. **Index Test**: AI methods and manual assessment were well-documented, ensuring consistent application.
3. **Flow and Timing**: Detailed workflow without exclusions or delays supports reliability.
4. **Recommendations**: Expand patient population to improve generalizability.

| **Domain** | **Risk of Bias** | **Applicability** |
| --- | --- | --- |
| **Patient Selection** | Moderate | Moderate |
| **Index Test/Ref Standard** | Low | High |
| **Flow and Timing** | Low | High |
| **Overall Bias** | Moderate | High |

# An Application for Wound Diagnosis and Treatment

1. **Patient Selection**: Dataset drawn from a specific region (Brazil), limiting broader applicability.
2. **Index Test**: CNN-based wound classification provided robust results, validated through appropriate metrics.
3. **Flow and Timing**: Clear methodology with no notable exclusions.
4. **Recommendations**: Incorporate datasets from diverse geographic regions for better applicability.

| **Domain** | **Risk of Bias** | **Applicability** |
| --- | --- | --- |
| **Patient Selection** | Moderate | Moderate |
| **Index Test/Ref Standard** | Low | High |
| **Flow and Timing** | Low | High |
| **Overall Bias** | Moderate | High |

# An Enhanced Diabetic Foot Ulcer Classification Approach Using GLCM and CNN

1. **Patient Selection**: Comprehensive dataset with balanced representation of wound types.
2. **Index Test**: Combined CNN and GLCM features yielded robust classification results.
3. **Flow and Timing**: No timing issues or exclusions; study design enhances reliability.
4. **Recommendations**: Validate on datasets covering different wound categories.

| **Domain** | **Risk of Bias** | **Applicability** |
| --- | --- | --- |
| **Patient Selection** | Low | High |
| **Index Test/Ref Standard** | Low | High |
| **Flow and Timing** | Low | High |
| **Overall Bias** | Low | High |

# Diabetic Foot Ulcer Segmentation Using Logistic Regression and DBSCAN Clustering

1. **Patient Selection**: Dataset restricted to a single region (Cuba) limits generalizability.
2. **Index Test**: Thorough segmentation pipeline demonstrated strong performance.
3. **Flow and Timing**: Minimal exclusions and well-defined methodology.
4. **Recommendations**: Increase diversity of datasets for broader application.

| **Domain** | **Risk of Bias** | **Applicability** |
| --- | --- | --- |
| **Patient Selection** | Moderate | Moderate |
| **Index Test/Ref Standard** | Low | High |
| **Flow and Timing** | Low | High |
| **Overall Bias** | Moderate | High |

# Automated Detection of Infection in Diabetic Foot Ulcer Images Using CNN

1. **Patient Selection**: Dataset limitations due to restricted access might affect reproducibility.
2. **Index Test**: CNN implementation demonstrated strong performance metrics.
3. **Flow and Timing**: Limited details on preparation steps and time intervals between stages.
4. **Recommendations:** Improve dataset accessibility and document preparation processes comprehensively.

| **Domain** | **Risk of Bias** | **Applicability** |
| --- | --- | --- |
| **Patient Selection** | Moderate | High |
| **Index Test/Ref Standard** | Low | High |
| **Flow and Timing** | Moderate | High |
| **Overall Bias** | Moderate | High |

# Recognition of Ischaemia and Infection in Diabetic Foot Ulcers: A Deep Convolutional Neural Network-Based Approach

**1. Patient Selection:**The study utilized a dataset specifically designed for diabetic foot ulcer (DFU) analysis, focusing on ischaemia and infection. The dataset included 1,459 images, which were augmented to provide balanced classes. Although the dataset's acquisition process followed standard protocols, limitations in diversity and lighting conditions could introduce bias.

**2. Index Test:**The study proposed a deep convolutional neural network (CNN) architecture named ResKNet, with variants like Res4Net and Res7Net. The CNN used augmented image patches resized to 128 × 128 for classification. The ischaemia recognition achieved outstanding performance with an AUC of 0.9968, while infection recognition showed an AUC of 0.8890 using Res7Net. The application of batch normalization and LeakyReLU activation optimized the learning process.

**3. Flow and Timing:**The dataset was split into training (70%), validation (10%), and testing (20%), ensuring all images were analyzed. However, the fixed image size and reliance on GPU-constrained environments led to potential compromises in resolution and detailed feature analysis.

**4. Recommendations:**

- Increase dataset diversity by including more lighting conditions and demographic variation.
- Apply higher-resolution images and test performance with different CNN architectures.
- Explore generative adversarial networks (GANs) for synthetic data augmentation.
- Conduct external validation to ensure the model's generalizability.

**Summary Table**

| **Domain** | **Risk of Bias** | **Applicability** |
| --- | --- | --- |
| **Patient Selection** | Moderate | High |
| **Index Test** | Low | High |
| **Flow and Timing** | Moderate | High |
| **Overall Bias** | Moderate | High |

# Predictive Risk Models for Wound Infection-Related Hospitalization or ED Visits in Home Health Care Using Machine Learning Algorithms

1. **Patient Selection**: Risk factors derived from clinical notes and structured data enhance predictive accuracy but limit generalizability beyond home healthcare settings.
2. **Index Test**: Comprehensive use of Logistic Regression, Random Forest, and ANN ensures methodological rigor.
3. **Flow and Timing**: Clear use of datasets spanning a complete home health care cycle.
4. **Recommendations**: Incorporate additional healthcare contexts to validate the predictive models.

| **Domain** | **Risk of Bias** | **Applicability** |
| --- | --- | --- |
| **Patient Selection** | Moderate | High |
| **Index Test/Ref Standard** | Low | High |
| **Flow and Timing** | Low | High |
| **Overall Bias** | Moderate | High |

**Qualitative Assessment:**

# Detect-and-Segment: A Deep Learning Approach to Automate Wound Image Segmentation

1. **Patient Selection**:
   - **Assessment**: The study utilized multiple independent datasets, including diabetic foot ulcers, systemic sclerosis digital ulcers, and various other wound types, ensuring diversity in data. However, the patient population may not fully represent all ethnicities or skin types.
   - **Limitations**: Limited representation of diverse demographics and wound conditions.
2. **Index Test**:
   - **Assessment**: The study employed a robust segmentation model combining detection and segmentation techniques with deep learning architectures like U-Net, ConvNet, DeepLab, and FCN. However, the segmentation models heavily relied on pre-trained architectures, and manual validation was minimal.
   - **Limitations**: Dependency on pre-trained models may affect adaptability to entirely new datasets.
3. **Flow and Timing**:
   - **Assessment**: The segmentation models were trained and tested on temporally and spatially diverse datasets. However, the precise timing of image collection relative to clinical procedures was not detailed, which might influence applicability in time-sensitive conditions.
   - **Limitations**: Inconsistent temporal data details.
4. **Risk of Bias and Applicability**:
   - **Assessment**: The study identified potential biases in wound detection accuracy, such as false positives or negatives in bounding box predictions. Despite these challenges, the method showed robustness in handling unseen data.
   - **Recommendations**: Include diverse datasets with varied lighting and skin tones. Implement real-time validation mechanisms to minimize detection errors.

| **Domain** | **Risk of Bias** | **Applicability** |
| --- | --- | --- |
| **Patient Selection** | Moderate | High |
| **Index Test** | Moderate | High |
| **Flow and Timing** | Low | High |
| **Overall Bias** | Moderate | High |

**Recommendations for Improvement:**

1. **Broader Dataset Inclusion**: Incorporate a more diverse range of skin tones, wound types, and clinical settings to enhance generalizability.
2. **Temporal Context**: Include details on the timing of image acquisition relative to wound progression to strengthen clinical relevance.
3. **Validation Studies**: Conduct additional validation studies involving real-world clinical workflows and user feedback.
4. **Bias Mitigation**: Implement mechanisms like feedback loops during image acquisition to minimize detection errors.

# Toward Machine-Learning-Based Decision Support in Diabetes Care

1. **Patient Selection**:
   Large, comprehensive dataset (246,705 patients) from Danish registers. Strong representativeness, though limited by potential bias due to missing historical data.
2. **Index Test**:
   Machine learning models (Random Forest, Logistic Regression) analyzed clinical and socio-economic data. Lacked blinding procedures for validation.
3. **Flow and Timing**:
   Well-documented follow-up durations for DFU (8 years) and amputation (6 years). Time between test application and outcome confirmation not explicitly detailed.
4. **Recommendations**:
   - Include blinded validation for prediction models.
   - Expand datasets from other healthcare systems.
   - Detail timing between test application and reference confirmation.

| **Domain** | **Risk of Bias** | **Applicability** |
| --- | --- | --- |
| Patient Selection | Moderate | High |
| Index Test | Moderate | High |
| Flow and Timing | Low | High |
| Overall Bias | Moderate | High |

# Exploiting Machine Learning Algorithms to Diagnose Foot Ulcers in Diabetic Patients

1. **Patient Selection**: Dataset and inclusion criteria are appropriate for the study's aim. No evidence of bias in patient selection.
2. **Index Test (ML Algorithms)**: Algorithms and evaluation metrics are robust and relevant for DFU prediction.
3. **Reference Standard**: Lack of clarity on how DFU ground truth labels were assigned raises concerns about reliability.
4. **Flow and Timing**: Clear and standard ML workflow with no exclusions or timing issues.

| **Domain** | **Risk of Bias** | **Applicability** |
| --- | --- | --- |
| Patient Selection | Low | Low |
| Index Test | Low | Low |
| Reference Standard | High | Uncertain |
| Flow and Timing | Low | Low |

**Recommendations:**

- Improve clarity on reference standard labeling.
- Expand dataset size and diversity.
- Validate algorithms prospectively in clinical settings.
- Enhance prediction explainability for clinical use.

# Machine Learning Models for Synthesizing Actionable Care Decisions on Lower Extremity Wounds

- 1. **Patient Selection**: Inclusion of diverse chronic wound types (e.g., diabetic foot, pressure ulcers); minimal bias.
  2. **Index Test**: Robust use of ML classifiers (e.g., XGBoost, SVM) and text features for decision-making.
  3. **Reference Standard**: Limited details on how decisions were labeled (expert consensus lacks external validation).
  4. **Flow and Timing**: Consistent workflow; no major exclusions.

**Recommendations**:

- - Enhance ground truth labels with more external validation.
  - Broaden dataset size and diversity for better generalizability.

| **Domain** | **Risk of Bias** | **Applicability** |
| --- | --- | --- |
| Patient Selection | Low | Low |
| Index Test | Low | Low |
| Reference Standard | High | Uncertain |
| Flow and Timing | Low | Low |

# Integrating 3D Model Representation for an Accurate Non-Invasive Assessment of Pressure Injuries with Deep Learning

- 1. **Patient Selection**: Includes diverse pressure injury stages; well-documented inclusion criteria.
  2. **Index Test**: Mask-RCNN with 2D/3D segmentation is robust and clinically relevant.
  3. **Reference Standard**: High-quality manual measurements used as ground truth.
  4. **Flow and Timing**: Transparent and reproducible process without exclusions.

**Recommendations**:

- - Evaluate the proposed method in larger datasets or real-world scenarios to validate scalability.

| **Domain** | **Risk of Bias** | **Applicability** |
| --- | --- | --- |
| Patient Selection | Low | Low |
| Index Test | Low | Low |
| Reference Standard | Low | Low |
| Flow and Timing | Low | Low |

# Pressure Ulcer Injury in Unstructured Clinical Notes: Detection and Interpretation

- 1. **Patient Selection**: Data from MIMIC-III ICU dataset; appropriate inclusion/exclusion criteria.
  2. **Index Test**: Innovative negation-aware processing for unstructured notes; enhances PUI detection.
  3. **Reference Standard**: Relies on ICD-9 codes and note-based keywords; potential for misclassification.
  4. **Flow and Timing**: Sufficient time for note aggregation and consistent methodology.

**Recommendations**:

- - Improve labeling accuracy by cross-validating ICD-9 codes and notes with clinical expert reviews.
  - Consider adding prospective data for better real-time applicability.

| **Domain** | **Risk of Bias** | **Applicability** |
| --- | --- | --- |
| Patient Selection | Low | Low |
| Index Test | Low | Low |
| Reference Standard | High | Uncertain |
| Flow and Timing | Low | Low |

# An Integrated Design for Classification and Localization of Diabetic Foot Ulcer Based on CNN and YOLOv2-DFU Models"

**Key Findings:**

- A 16-layer CNN was designed for classification of DFU images into ischemia/infection categories, achieving high sensitivity, specificity, and accuracy.
- YOLOv2-DFU network localized infected regions effectively using ShuffleNet as its backbone.
- The methodology was validated on a newly developed dataset, outperforming existing models in precision and accuracy.

**Recommendations:**

- Include external validation datasets to confirm generalizability.
- Evaluate the framework on real-world clinical settings for broader applicability.

| **Domain** | **Risk of Bias** | **Applicability** |
| --- | --- | --- |
| Patient Selection | Low | Low |
| Index Test | Low | Low |
| Reference Standard | Low | Low |
| Flow and Timing | Low | Low |

# Robust Methods for Real-Time Diabetic Foot Ulcer Detection and Localization on Mobile Devices

**Key Findings:**

- A robust CNN model using Faster R-CNN with InceptionV2 achieved high precision and speed for DFU localization.
- Two-tier transfer learning demonstrated efficacy, while the model was optimized for mobile platforms (Jetson TX2, Android).
- The study relied on a comprehensive dataset of 1775 annotated images.

**Recommendations:**

- Expand the dataset to include more diverse and challenging cases.
- Conduct longitudinal studies to evaluate the model's real-time performance in clinical scenarios.

| **Domain** | **Risk of Bias** | **Applicability** |
| --- | --- | --- |
| Patient Selection | Low | Low |
| Index Test | Low | Low |
| Reference Standard | Low | Low |
| Flow and Timing | Low | Low |

# Computational Approach for Chronic Wound Tissue Characterization

**Key Findings:**

- Fuzzy c-means clustering combined with machine learning classifiers (LDA, DT, RF) achieved high accuracy for chronic wound segmentation.
- The approach showed promise with an accuracy of 93.75% compared to clinician-grounded evaluations.
- The segmentation and classification process effectively leveraged histogram sampling and color feature enhancement.

**Recommendations:**

- Incorporate a larger dataset with diverse wound types to enhance robustness.
- Implement prospective evaluations to assess performance in real-world scenarios.

| **Domain** | **Risk of Bias** | **Applicability** |
| --- | --- | --- |
| Patient Selection | Low | Low |
| Index Test | Low | Low |
| Reference Standard | Low | Low |
| Flow and Timing | Low | Low |

# Segmentation and Measurement of Chronic Wounds for Bioprinting

**Key Findings:**

- Livewire segmentation outperformed other methods with 97.08% accuracy and high specificity for delineating chronic wounds.
- The integration of segmentation results into a bioprinter robot demonstrated practical applications in wound healing.
- Achieved 95.56% similarity between bioprinted patches and wound geometry.

**Recommendations:**

- Expand datasets with larger image pools for better training and validation.
- Validate the bioprinting pipeline in diverse clinical settings to assess scalability.

| **Domain** | **Risk of Bias** | **Applicability** |
| --- | --- | --- |
| Patient Selection | Low | Low |
| Index Test | Low | Low |
| Reference Standard | Low | Low |
| Flow and Timing | Low | Low |

# Tissue Classification and Segmentation of Pressure Injuries Using Convolutional Neural Networks

**Key Findings:**

- CNN-based segmentation of pressure injuries achieved 92.01% accuracy and high precision for granulation and necrotic tissue classification.
- Preprocessing steps (flashlight removal and sub-image extraction) ensured robust segmentation of small, diverse tissue regions.
- The dataset included 22 high-resolution images with manual ground-truth annotations.

**Recommendations:**

- Expand the dataset to include a wider range of pressure injury stages and patient demographics.
- Assess the model's performance on low-resource devices to enhance clinical utility.

| **Domain** | **Risk of Bias** | **Applicability** |
| --- | --- | --- |
| Patient Selection | Low | Low |
| Index Test | Low | Low |
| Reference Standard | Low | Low |
| Flow and Timing | Low | Low |

# Non-invasive Method to Analyze the Risk of Developing Diabetic Foot"

**Key Findings:**

- The study developed a non-invasive method using K-means clustering to classify diabetic patients into high and low risk for foot ulcers based on self-care data.
- Achieved 97% accuracy with simulated data but only 68% with real data when validated against clinical expert classifications.

**Recommendations:**

- Increase dataset diversity and size for real-world applicability.
- Validate the model prospectively in clinical settings to improve classification reliability.

| **Domain** | **Risk of Bias** | **Applicability** |
| --- | --- | --- |
| Patient Selection | Low | Low |
| Index Test | Low | Low |
| Reference Standard | High | Uncertain |
| Flow and Timing | Low | Low |

# Classification of Pressure Ulcer Tissues with 3D Convolutional Neural Network

**Key Findings:**

- Proposed a 3D CNN framework for segmenting pressure ulcer tissues into granulation, necrotic, and slough regions.
- Achieved high performance (AUC = 95%, Dice Coefficient = 92%) on a dataset of 193 annotated images.

**Recommendations:**

- Validate the method on larger, more diverse datasets.
- Consider implementing real-time application capabilities for clinical settings.

| **Domain** | **Risk of Bias** | **Applicability** |
| --- | --- | --- |
| Patient Selection | Low | Low |
| Index Test | Low | Low |
| Reference Standard | Low | Low |
| Flow and Timing | Low | Low |

# An Image-Based Object Recognition System for Wound Detection and Classification of Diabetic Foot and Venous Leg Ulcers

**Key Findings:**

- The YoloV5m6 model achieved high precision (0.942) and recall (0.837) for classifying diabetic foot and venous leg ulcers from 885 annotated wound images.
- Demonstrated robustness with augmentation techniques to address dataset limitations.

**Recommendations:**

- Expand datasets to include more diverse wound characteristics and patient demographics.
- Integrate the model into clinical workflows with user-friendly dashboards for clinician validation.

**Summary Table:**

| **Domain** | **Risk of Bias** | **Applicability** |
| --- | --- | --- |
| Patient Selection | Low | Low |
| Index Test | Low | Low |
| Reference Standard | Low | Low |
| Flow and Timing | Low | Low |

# Efficient Detection of Wound-Bed and Peripheral Skin with Statistical Colour Models

**Key Findings:**

- Developed statistical color models for segmenting wound tissues (granulation, necrosis, slough) with high accuracy (AUC = 94.26%).
- Validated on a diverse dataset of 435 pressure ulcer images with expert annotations.

**Recommendations:**

- Explore integration with portable devices for field applications.
- Extend to multi-skin tone datasets to ensure generalizability.

| **Domain** | **Risk of Bias** | **Applicability** |
| --- | --- | --- |
| Patient Selection | Low | Low |
| Index Test | Low | Low |
| Reference Standard | Low | Low |
| Flow and Timing | Low | Low |
